# Body size, functional form and environmental correlates of maximum longevity in marine‐occurring fishes

**DOI:** 10.1111/jfb.70629

**Published:** 2026-08-30

**Authors:** Longyu Li, Zenghui Xu

**Affiliations:** ^1^ Graduate School of Frontier Sciences (GSFS), The University of Tokyo Chiba Japan; ^2^ Ocean College Zhejiang University Zhejiang China

**Keywords:** body size, FishBase, FishTree, longevity, maximum depth, quantile regression

## Abstract

Maximum longevity varies widely among marine‐occurring fishes, but the form of its relationship with body size and the contribution of broad environmental gradients remain uncertain. We analysed 757 species, using maximum length as the primary size metric and completed mass only in sensitivity analyses. A quadratic length model improved fit over a linear comparator and captured broad attenuation in the size–longevity association. Maximum depth was positively associated with log10 maximum longevity after accounting for this curvature (estimate per 1000 m = 0.0692, 95% CI 0.0312 to 0.1072, *p* < 0.001). Latitude estimates varied among range‐based metrics and model specifications, and the latitude term performed less consistently when entire families and orders were withheld. None of the group or continuous‐interaction contrasts met the defined FDR threshold of *q* < 0.05. FishTree matched 605 species but excluded every elasmobranch in the canonical dataset; branch‐length phylogenetic generalized least squares (PGLS) therefore provides a matched‐subset sensitivity rather than full phylogenetic coverage. Linear contrasts from 5000 species‐level bootstrap resamples and nonlinear quantile sensitivities did not indicate broad upper‐tail steepening. Teleostei‐only, broad‐Class‐adjusted and Family‐clustered analyses retained a positive maximum‐depth coefficient, whereas latitude was less stable under clustered uncertainty. Body size and maximum depth thus provide the most consistent macroecological associations with maximum longevity. Latitude remains exploratory, and the data do not identify causal mechanisms.

## 1 INTRODUCTION

Maximum longevity integrates growth, maturation and mortality schedules over an organism's lifespan. Body size is often among its strongest comparative correlates because size covaries with growth, energetic demand, predation exposure and demographic turnover. Yet broad comparisons also show substantial variation in ageing and survival among lineages (Brown et al., 2004; Charnov, 1993; Jones et al., 2014; Stearns, 1992). A comparative size‐longevity analysis must therefore estimate the form of the relationship instead of assuming that one exponent applies across the full size range.

Marine‐occurring fishes provide a stringent test because they span several orders of magnitude in size and include unusually long‐lived taxa such as the Greenland shark *Somniosus microcephalus* and Pacific rockfishes *Sebastes* spp. (Kolora et al., 2021; Nielsen et al., 2016). Fish life histories link size, growth and mortality, but body size cannot by itself represent thermal exposure, oxygen availability, ecological mortality or sampling history (Andersen et al., 2016; Charnov & Gillooly, 2004; Gislason et al., 2010; Stark, 2022). Maximum‐age records also depend on ageing method and validation quality, especially among long‐lived species (Campana, 2001).

Maximum depth and latitude summarize broad environmental gradients. Greater depth can coincide with lower temperature, reduced light and different resource or predation regimes, whereas latitude covaries with temperature, seasonality and biogeographic history (Black et al., 2021; Drazen & Haedrich, 2012; Munch & Salinas, 2009; Pauly, 1980; Priede & Froese, 2013; Rubalcaba et al., 2020). These variables do not identify the operative pathway. Extrinsic mortality and ageing may be related in several ways, and species‐level trait databases do not resolve local mortality schedules (Dańko et al., 2018; de Vries et al., 2023). We therefore evaluate depth and latitude as macroecological correlates.

Ordinary least squares (OLS) describes conditional‐mean associations, whereas quantile regression tests whether fitted slopes differ across the observed response distribution (Cade & Noon, 2003; Koenker, 2005; Koenker & Bassett, 1978). Visually prominent scatterplot edges require formal contrasts before they can be interpreted as distributional structure (Scharf et al., 1998). Phylogenetic generalized least squares (PGLS) offers a complementary matched‐subset analysis by modelling residual covariance under a specified tree and branch‐length structure (Felsenstein, 1985; Freckleton et al., 2002; Symonds & Blomberg, 2014).

We asked five questions. Is the size‐longevity relationship adequately represented by a linear model, or does a low‐complexity nonlinear form fit better? Do maximum depth and a range‐based latitude proxy add to the retained size relationship? Do diagnostic group slopes or continuous interactions indicate variation in the centred length coefficient? Are broad associations consistent in the FishTree‐matched subset and across taxonomic sensitivities? Do linear and nonlinear quantile analyses indicate steeper upper‐tail relationships? Length‐based analyses were primary because their provenance was more direct; mass‐based models were sensitivity evidence.

## 2 METHODS

### 2.1 Data assembly, filtering and trait provenance

We assembled the canonical dataset from FishBase release 25.04 queries archived through rfishbase (Boettiger et al., 2012; Froese & Pauly, 2026). The FishBase web resource and species‐name query were accessed on 10 June 2026; signed stock endpoints and maximum‐length‐type metadata were queried or refreshed on 13 July 2026. The executable filtering sequence began with 36,132 species records, retained 18,666 with Saltwater = 1, 16,942 with positive maximum length, 930 with positive maximum longevity, 833 with maximum depth and 757 with both latitude endpoints; identifier and recovered‐name checks left the same 757‐species canonical dataset. Thus, 17,466, 1724, 16,012, 97 and 76 species were excluded at the successive substantive filters. The archived species table already contained one row per SpecCode. Repeated stock records were deterministically summarized within SpecCode before the one‐to‐one join; signed northern and southern limits were the maximum and minimum reconstructed endpoints, respectively, and fail‐fast uniqueness checks stopped processing if any SpecCode remained duplicated. Complete‐case filtering was applied to longevity, length, depth and latitude, and these primary fields were not imputed. Harmonized species‐level metadata on ageing methods and validation status were not consistently available, so no species were excluded on this basis; the associated uncertainty is addressed in Discussion 4.5. Longevity was recorded in years, length in centimetres, mass in grams, depth in metres and latitude in degrees; longevity, length and mass were log10‐transformed for modelling. Further details on dataset assembly, variable definitions and provenance are provided in Section S1, Figure S1 and Table S1.

Maximum length was the primary size variable. FishBase identified total length (TL) for 595 species, standard length for 119, fork length for 35 and other or unspecified length types for eight. Length types were not converted because species‐specific conversion factors were unavailable. This heterogeneity may introduce measurement error, so a TL‐only sensitivity analysis was used to assess its influence. The complete length‐type inventory and TL‐only sensitivity are reported in Table S3.

Completed mass was used only in sensitivity analyses. Direct body‐mass records were available for 424 species. For 261 species, mass was reconstructed using

$$W=aL^{b}$$

where *W* is mass in grams, *L* is length in centimetres and *a* and *b* are arithmetic means of the available FishBase length–weight coefficients within SpecCode. No genus‐level coefficients were substituted. A further 72 completed mass values used the archived fallback

$$W=0.01L^{3}$$

These 72 entries were classified as unresolved in provenance because their values could not be linked to a direct mass record or species‐level coefficient records. This fallback, together with the strong collinearity between length and mass, precluded interpretation of mass as an independent primary predictor. Completed‐mass provenance is summarized in Table S2.

### 2.2 Environmental predictors, latitude reconstruction, phylogeny and data support

Maximum depth was analysed continuously per 1000 m. Signed latitude endpoints were reconstructed from the NorthSouthN and NorthSouthS hemisphere fields in the FishBase stocks table: the northern limit was the largest signed endpoint and the southern limit the smallest. Absolute range‐midpoint latitude was calculated as the absolute value of the midpoint between the signed northern and southern range limits:

$$\left|\frac{\text{northern limit}+\text{southern limit}}{2}\right|$$

It was analysed per 10°. Latitudinal range width and maximum absolute endpoint were retained for sensitivities; 301 species ranges crossed the equator. Alternative models used the legacy mean absolute endpoint proxy, the maximum absolute endpoint, the signed midpoint after excluding cross‐equatorial species and midpoint together with range width. The latitude variable is a range‐based proxy rather than an occurrence‐weighted geographical or thermal exposure measure.

Continuous depth and latitude were used for primary environmental inference. Categories described support and summarized diagnostic slopes: epipelagic‐associated (<200 m), mesopelagic‐associated (200–<1000 m) and bathypelagic‐associated (≥1000 m) for depth, and low (<23.5°), intermediate (23.5–66.5°) and high (>66.5°) absolute range‐midpoint latitude. Latitude groups contained 314, 438 and five species, respectively, so the high group was descriptive. Pairwise predictor‐overlap plots, group‐wise length distributions, depth‐by‐latitude cell counts and length‐bin occupancy were examined before grouped comparisons (Zuur et al., 2010). Common‐support subsets excluded the high group, restricted analyses to the intermediate group, retained the observed shared length range among depth groups, or retained the intersection of their 5th–95th percentile length ranges. Predictor overlap and length‐bin occupancy are reported in Section S5 and Table S18.

Scientific names were queried using the fishtree R package against the FishTree ray‐finned fish phylogenetic resource; 605 of 757 species matched and 152 unmatched species were excluded from phylogenetic models without grafting (Chang et al., 2019; Rabosky et al., 2018). The returned 605‐tip chronogram required only a numerical terminal‐branch correction within 7 × 10^−6^ branch‐length units to satisfy ultrametric tolerance; topology was unchanged, and the corrected tree was used for PGLS. Full diagnostics are provided in Table S5. The FishTree‐matched subset was dominated by ray‐finned fishes and excluded all elasmobranch species in the canonical dataset: it contained 595 Teleostei and 10 Chondrostei, whereas all 75 Elasmobranchii, three Petromyzonti and one Coelacanthi were unmatched. Only three of the five high range‐midpoint species matched. FishTree matching, taxonomic coverage and chronogram diagnostics are detailed in Section S2 and Table S4.

### 2.3 Primary models and functional‐form assessment

Let y denote log10 maximum longevity, *x* log10 maximum length, *xc* = *x* − mean(*x*), *xc* ^2^ the squared centred length term, depth1000 = maximum depth/1000 and latitude10 = absolute range‐midpoint latitude/10. We compared *y* ~ *xc* with *y* ~ *xc* + *xc* ^2^ and fitted *y* ~ *s*(*xc*, *k* = 4) as a low‐complexity generalized additive model (GAM; Wood, 2017). An ML‐fitted GAM provided an Akaike information criterion (AIC) sensitivity on the same observations and Gaussian likelihood, whereas a REML‐fitted GAM was used only as a flexible shape sensitivity and was not ranked against the linear models by AIC. In the final reanalysis, functional form was evaluated using a model‐evaluation framework that considered AIC improvement, the nested‐model test, reduction in systematic residual trend and stability of the curvature term within central common support. The quadratic was retained because its AIC was at least 10 units lower than that of the linear model, the nested F test had *p* < 0.01, the RMS of the fitted residual trend fell by at least 50% and the quadratic coefficient retained its direction in central common support. The *xc* coefficient is the local slope near mean log10 length. The quadratic is a parsimonious approximation to broad attenuation, not an exact second‐order biological law.

The primary environmental OLS model was *y* ~ *x*_c + *x*_c2 + depth_1000 + latitude_10. Depth‐only, alternative‐latitude, TL‐only, mass and taxonomic‐composition models were supplementary sensitivities. Group models retained common curvature and added x_c‐by‐group interactions. Continuous‐interaction models added x_c‐by‐depth and x_c‐by‐latitude terms using raw depth and log10(maximum depth + 1 m) in parallel. Common‐support subsets were refitted with the primary additive model. Benjamini‐Hochberg FDR adjustment was applied within the defined depth‐contrast, latitude‐contrast, continuous‐interaction and quantile‐contrast families (Benjamini & Hochberg, 1995).

For linear, quadratic and GAM fits, residual RMSE and the RMS of a loess‐smoothed residual trend were computed separately. The same residual‐pattern function used fitted values, model residuals, span 0.75 and degree 2 for every form; only the RMS of the fitted residual trend informed the curvature diagnostic. Residual‐versus‐fitted plots, Q–Q plots, Cook's distances and a Breusch–Pagan‐style auxiliary regression of squared residuals on fitted values were also examined. Observations with Cook's distance greater than 4/n were flagged for influence assessment, but this diagnostic threshold did not trigger automatic exclusion; all primary analyses retained the complete canonical dataset unless otherwise stated. Predictor overlap and collinearity were assessed before interpretation (Dormann et al., 2013). The fitted quadratic derivative reached zero near 487 cm, but only 15 species occurred above that point and it lay outside the central common‐support window. This location was not treated as a biological threshold, the descending extreme tail was not extrapolated beyond the observed range and its shape was considered poorly resolved. Collinearity diagnostics are provided in Table S17 and Figure S4.

### 2.4 Phylogenetic, quantile and validation analyses

PGLS models used the same centred quadratic form and environmental display scales as OLS, with residual covariance derived from the numerically corrected FishTree chronogram using ape and caper (Orme et al., 2025; Paradis & Schliep, 2019). Pagel's λ was estimated by maximum likelihood for each primary model. Grouped PGLS models fixed *λ* at the primary environmental estimate for their contrasts; a supplementary analysis estimated *λ* independently for each grouped model. PGLS is therefore a matched‐subset phylogenetic‐covariance sensitivity, not a correction representing all 757 canonical species.

Linear quantile regressions were fitted separately for length and mass at *τ* = 0.10, 0.25, 0.50, 0.75, 0.90 and 0.95 using quantreg::rq. Species‐level bootstrap contrasts used *B* = 5000 resamples with replacement and seed 20,260,610; OLS and quantile slopes were refitted in every resample. Percentile 95% intervals were calculated for *β*_0.90_ − *β*_0.50_, *β*₀.₉₅ − *β*_0.50_, *β*_0.90_ − *β*_OLS_ and *β*₀.₉₅ − *β*_OLS_. Two‐sided bootstrap p‐values were calculated as

$$p=2\times \min\left\{\mathrm{Pr}\left(\Delta \leq 0\right),\mathrm{Pr}\left(\Delta \geq 0\right)\right\}$$

where Δ denotes the resampled contrast; values were capped at 1. Quadratic quantile models at *τ* = 0.50, 0.90 and 0.95 used *B* = 1000 species bootstraps and seed 20,260,713; predicted curves and local slope differences at the 25th, 50th and 75th percentiles of log10 length were summarized with percentile intervals. These non‐phylogenetic analyses describe distributional variation in the canonical dataset.

Internal five‐fold diagnostics compared a nested sequence of quadratic length alone, quadratic length plus maximum depth and quadratic length plus maximum depth and the latitude proxy. For species‐random folds, unique SpecCode values were sorted in ascending order, permuted after set. seed(20260713) and allocated cyclically to five folds of 151 or 152 species. The archived map records scientific name, random order, fold, seed and assignment rule, and no species crossed folds. Family‐ and order‐blocked diagnostics kept each taxonomic group in one fold. Groups were ordered by decreasing species count; ties were randomized with seeds 20,260,713 and 20,260,813, respectively, and a greedy algorithm assigned each intact group to the currently smallest fold. No taxonomic label was missing. Whole‐order sizes produced necessarily unequal folds of 106–224 species. RMSE, MAE and predictive R^2^ are internal transportability diagnostics, not external measures of predictive accuracy.

The pooled held‐out coefficient of determination was calculated as follows:

Rpred2=1−∑iyi−y^i2∑iyi−ȳ2

Here, *y*_i_ is the held‐out observed response, *ŷ*_i_ is its held‐out prediction and *ȳ* is the mean of all pooled held‐out observations. Main‐text predictive *R* ^2^ values were calculated from the pooled held‐out predictions; fold‐level values and their standard deviations are reported in the Supporting Information. A negative predictive *R* ^2^ indicates performance below the pooled mean‐only comparator.

Taxonomic‐composition sensitivities refitted the final OLS model in Teleostei, added broad Class fixed effects to the canonical model, and estimated Family‐clustered CR2 standard errors with Satterthwaite degrees of freedom using clubSandwich. A 75‐species elasmobranch‐only model and predictor ranges were reported as supplementary support checks. Additional sensitivities compared the two classes represented in FishTree and the 605 matched species. These models do not replace the canonical OLS analysis or broaden the PGLS sample.

### 2.5 Software, reproducibility and reporting

Analyses were run in R 4.6.0 (R Core Team, 2026). Direct package versions included quantreg 6.1, ape 5.8‐1, caper 1.0.4, fishtree 0.3.4, rfishbase 5.0.3, mgcv 1.9‐4, clubSandwich 0.7.0, sandwich 3.1‐2 and ggplot2 4.0.3. Fixed seeds were 20,260,610 for linear quantile bootstraps, 20,260,713 for nonlinear quantile bootstraps and species/family fold ties and 20,260,813 for order‐fold ties. The reproducibility package contains canonical data, cached source queries, returned and corrected chronograms, scripts, fold maps, model outputs, figures, tables and session information.

Check mode validates required files and independently specified expected dimensions; missing sources, invalid statuses or mismatched values terminate execution. A negative control deliberately perturbs the expected canonical row count and must return FAIL. Full rebuild mode reruns the analysis and figure pipeline, performs dimension checks before and after analysis and records stdout, stderr and session information. Figures and tables are generated from machine‐readable outputs. Data and code are available at https://doi.org/10.5281/zenodo.21769695. Estimates and confidence intervals are displayed to four decimals in main tables, *p*‐values to three decimals or <0.001 and FDR *q*‐values to three decimals.

## 3 RESULTS

### 3.1 Dataset coverage and analysis sets

The canonical dataset contained 757 complete‐case species, of which 605 matched FishTree and 152 remained unmatched. Total length accounted for 595 maximum‐length records. Mass provenance comprised 424 raw values, 261 length‐weight derivations and 72 values with unresolved provenance. Depth groups contained 373, 273 and 111 species, whereas latitude groups contained 314 low, 438 intermediate and five high range‐midpoint species. Depth‐by‐latitude coverage was incomplete, and the five‐species high group was descriptive (Figure 1; Figure S5). Analysis‐set coverage, units and variable provenance are summarized in Table 1.

**FIGURE 1:**
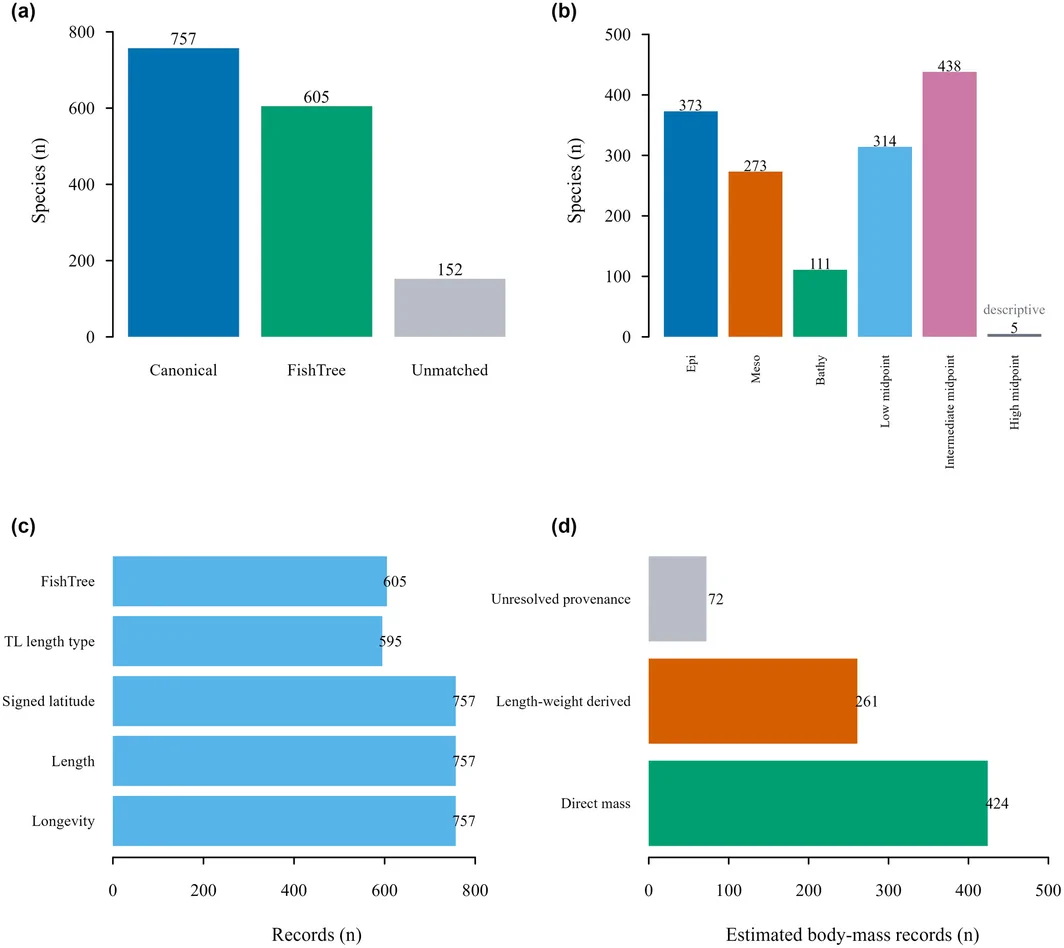
Dataset structure and variable provenance. (a) reports the canonical, FishTree‐matched and unmatched analysis sets. (b) shows depth and final range‐midpoint latitude group counts; the high range‐midpoint group (*n* = 5) is descriptive. (c) summarizes variable and length‐type coverage. (d) separates direct, length‐weight‐derived and unresolved body‐mass provenance. Mass‐based analyses are sensitivity evidence.

**TABLE 1:** Analysis‐set coverage, units and variable provenance.

Item	Coverage	Unit or definition	Analytical role
Canonical analysis dataset	757	Species	Primary OLS and quantile dataset
FishTree‐matched subset	605	Species	PGLS robustness subset
Unmatched species	152	Species	Documented; not grafted
Maximum longevity	757	Years; log10 transformed	Response
Maximum length	757	cm; log10 transformed	Primary size predictor
Maximum depth	757	m; displayed per 1000 m	Primary continuous environmental predictor
Signed latitude range endpoints	757	Degrees; absolute signed range midpoint, displayed per 10°	Primary latitude metric with metric‐sensitivity checks
Total‐length records	595	FishBase maximum‐length type TL	Length‐type sensitivity subset
Raw estimated body mass	424	g	Mass provenance
Length‐weight‐derived estimated body mass	261	g	Mass sensitivity only
Estimated body mass with unresolved provenance	72	g	Mass sensitivity only; no effect on length analyses

*Note*: Unresolved body‐mass provenance affects only mass sensitivity analyses. Depth and latitude groups are diagnostic strata.

Abbreviation: TL, total length.

FishTree coverage was taxonomically uneven. The matched subset contained 595 Teleostei and 10 Chondrostei, but none of the 75 Elasmobranchii, three Petromyzonti, or one Coelacanthi. These coverage differences bound the interpretation of PGLS and grouped comparisons.

### 3.2 Nonlinear body size‐longevity association

The size–longevity relationship was positive but not adequately summarized by one linear coefficient. The quadratic model had AIC = 497.486, a decrease of 43.345 units relative to the linear model (AIC = 540.831); adjusted *R* ^2^ increased from 0.317 to 0.356, and the RMS of the fitted residual trend decreased from 0.0971 to 0.0378. Its centred linear coefficient was 0.5812 (95% CI 0.5223 to 0.6402, *p* < 0.001) and the quadratic term was −0.3233 (95% CI −0.4163 to −0.2303, *p* < 0.001). The central common‐support estimate of the quadratic term remained negative (−0.9581, 95% CI −1.3016 to −0.6146, *p* < 0.001).

As a flexible shape sensitivity, the ML‐fitted GAM had AIC = 492.601 and an RMS of the fitted residual trend of 0.0375, while the REML fit described a similar broad attenuation. Neither fit was treated as a separate biological model. The quadratic derivative reached zero near 487 cm, beyond the central common‐support upper limit; 15 species lay above that location and five exceeded the Cook's distance diagnostic threshold. The quadratic therefore summarizes broad attenuation, whereas the descending extreme tail is poorly resolved and is not interpreted as evidence that longevity declines among the largest fishes (Figure 2; Table S6). Complete functional‐form and selected OLS outputs are provided in Section S3 and Table S7.

**FIGURE 2:**
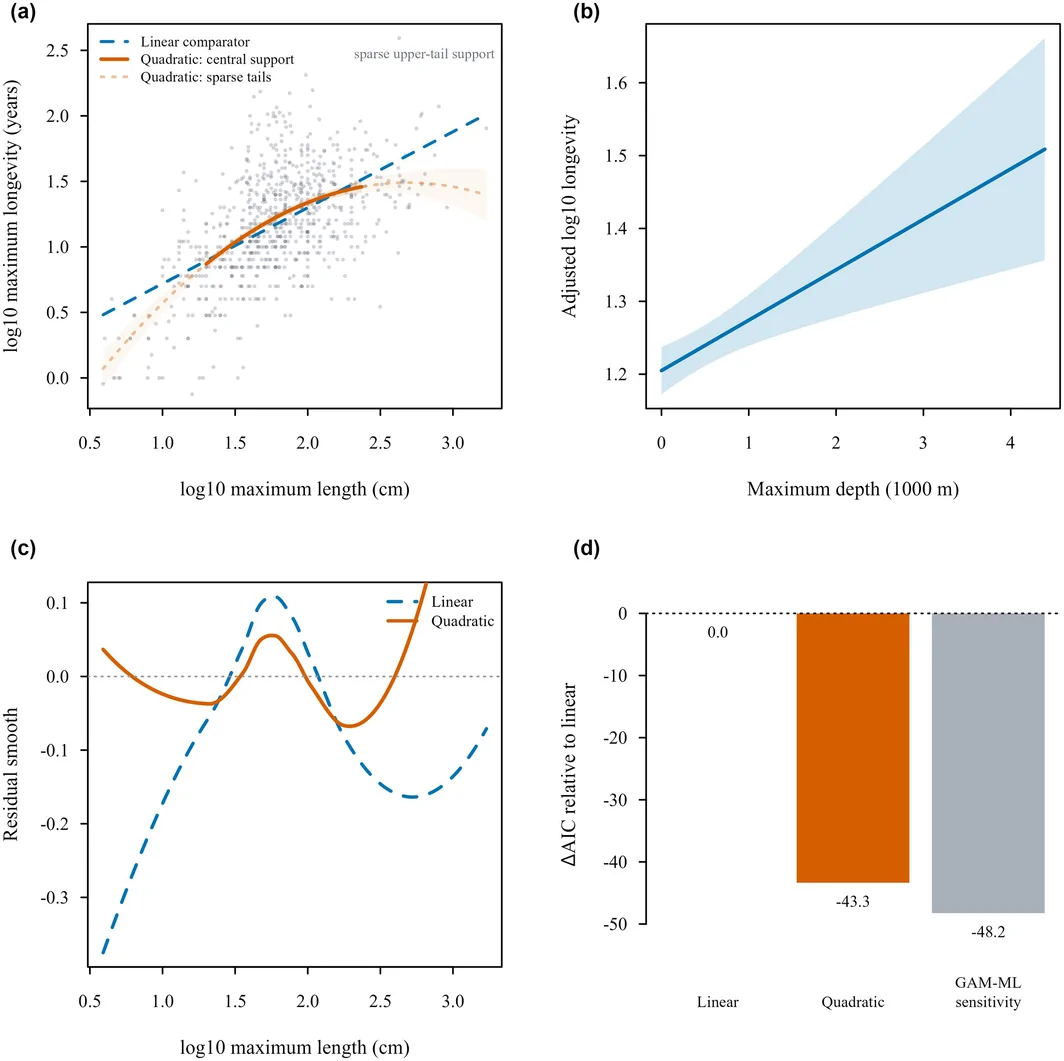
Functional form and environmental association. (a) compares the linear model with the retained quadratic approximation and its 95% confidence band. The quadratic is shown as a solid line within central common support and as a pale dashed segment in the sparse upper tail. (b) shows the adjusted maximum‐depth association at centred length and median absolute range‐midpoint latitude. (c) compares residual smooths from linear and quadratic size models. (d) shows AIC differences relative to the linear model for the quadratic model and the ML‐fitted generalized additive model (GAM) sensitivity; the REML‐fitted GAM was used only as a flexible shape sensitivity. The quadratic curve is used as a parsimonious approximation to broad attenuation. Its descending extreme tail lies outside central common support and is not interpreted as evidence that longevity declines among the largest fishes.

### 3.3 Environmental associations after accounting for curvature

Maximum depth showed the most consistent environmental association beyond size. In the primary OLS model, the estimate per 1000 m was 0.0692 (95% CI 0.0312 to 0.1072, *p* < 0.001), with adjusted *R* ^2^ = 0.379. On the original longevity scale, this estimate corresponds to approximately 17% higher expected maximum longevity per additional 1000 m of maximum depth, holding body size and the latitude proxy constant. The positive direction was retained in the central common‐support subset (0.1007, 95% CI 0.0575 to 0.1439, *p* < 0.001) and the intermediate‐latitude subset (0.0696, 95% CI 0.0265 to 0.1127, *p* = 0.002).

The absolute signed range‐midpoint latitude estimate was 0.0200 per 10° (95% CI 0.0078 to 0.0323, *p* = 0.001), but alternative range summaries were less consistent. The legacy mean absolute endpoint and maximum absolute endpoint intervals included zero, as did the stricter central common‐support estimate (0.0101, 95% CI −0.0034 to 0.0237, *p* = 0.143). Latitude was therefore retained as an exploratory association based on a range‐midpoint proxy, not as an environmental association comparable in stability to maximum depth (Table 2; Tables S20 and S21). Complete quadratic environmental‐model outputs are reported in Table S10.

**TABLE 2:** Final primary OLS and PGLS models with mass sensitivity analyses.

Analysis	Dataset	Term	Estimate	SE	95% CI	*p*	Adjusted *R* ^2^	AIC	Pagel's *λ*	Role
OLS size	Canonical, *n* = 757	Centered log10 length	0.5812	0.0300	0.5223 to 0.6402	<0.001	0.356	497.486		Primary OLS
OLS size	Canonical, *n* = 757	Centered log10 length squared	−0.3233	0.0474	−0.4163 to −0.2303	<0.001	0.356	497.486		Primary OLS
OLS environment	Canonical, *n* = 757	Centered log10 length	0.5764	0.0304	0.5168 to 0.6361	<0.001	0.379	471.693		Primary OLS
OLS environment	Canonical, *n* = 757	Centered log10 length squared	−0.3207	0.0471	−0.4132 to −0.2282	<0.001	0.379	471.693		Primary OLS
OLS environment	Canonical, *n* = 757	Maximum depth / 1000 m	0.0692	0.0194	0.0312 to 0.1072	<0.001	0.379	471.693		Primary OLS
OLS environment	Canonical, *n* = 757	Range‐midpoint latitude / 10°	0.0200	0.0062	0.0078 to 0.0323	0.001	0.379	471.693		Primary OLS
PGLS environment	FishTree, *n* = 605	Centered log10 length	0.6693	0.0439	0.5830 to 0.7555	<0.001	0.352	29.169	0.887	PGLS robustness
PGLS environment	FishTree, *n* = 605	Centered log10 length squared	−0.1397	0.0675	−0.2723 to −0.0070	0.039	0.352	29.169	0.887	PGLS robustness
PGLS environment	FishTree, *n* = 605	Maximum depth / 1000 m	0.1062	0.0225	0.0621 to 0.1503	<0.001	0.352	29.169	0.887	PGLS robustness
PGLS environment	FishTree, *n* = 605	Range‐midpoint latitude / 10°	0.0397	0.0081	0.0237 to 0.0557	<0.001	0.352	29.169	0.887	PGLS robustness
OLS mass sensitivity	Canonical, *n* = 757	log10 completed mass	0.2166	0.0104	0.1961 to 0.2371	<0.001	0.388	459.660		Sensitivity
OLS mass sensitivity	Canonical, *n* = 757	Maximum depth / 1000 m	0.0665	0.0189	0.0293 to 0.1036	<0.001	0.388	459.660		Sensitivity
OLS mass sensitivity	Canonical, *n* = 757	Range‐midpoint latitude / 10°	0.0296	0.0062	0.0175 to 0.0417	<0.001	0.388	459.660		Sensitivity
PGLS mass sensitivity	FishTree, *n* = 605	log10 completed mass	0.2131	0.0142	0.1852 to 0.2411	<0.001	0.336	44.338	0.871	Sensitivity
PGLS mass sensitivity	FishTree, *n* = 605	Maximum depth / 1000 m	0.1083	0.0226	0.0639 to 0.1528	<0.001	0.336	44.338	0.871	Sensitivity
PGLS mass sensitivity	FishTree, *n* = 605	Range‐midpoint latitude / 10°	0.0422	0.0082	0.0261 to 0.0583	<0.001	0.336	44.338	0.871	Sensitivity

*Note*: Length is centred at mean log10 maximum length = 1.7882. The squared term represents curvature. Maximum depth and absolute signed range‐midpoint latitude are displayed per 1000 m and 10°. PGLS applies to 605 FishTree‐matched species. Mass models are sensitivity analyses. AIC values are comparable only among models fitted to the same response observations and likelihood framework. Values from OLS and PGLS models, or from models fitted to different analysis subsets, should not be compared directly.

### 3.4 Diagnostic group interactions and taxonomic sensitivity

None of the common‐curvature group contrasts met the defined FDR threshold of *q* < 0.05. OLS depth contrasts had q‐values of 0.335–0.498, and the low‐minus‐intermediate latitude contrast was −0.1134 (95% CI −0.2369 to 0.0101, *p* = 0.072, *q* = 0.216). The high group contained five species and its contrasts were neither estimated nor interpreted. Continuous interactions followed the same hierarchy: two terms had unadjusted *p*‐values below 0.05 under log‐depth scaling, but none met the defined FDR threshold; all *q*‐values were at least 0.095 (Figures 3 and 4; Table 3). Complete OLS group‐slope contrasts are reported in Section S4 and Table S11. Continuous‐interaction sensitivities are reported in Table S19.

**FIGURE 3:**
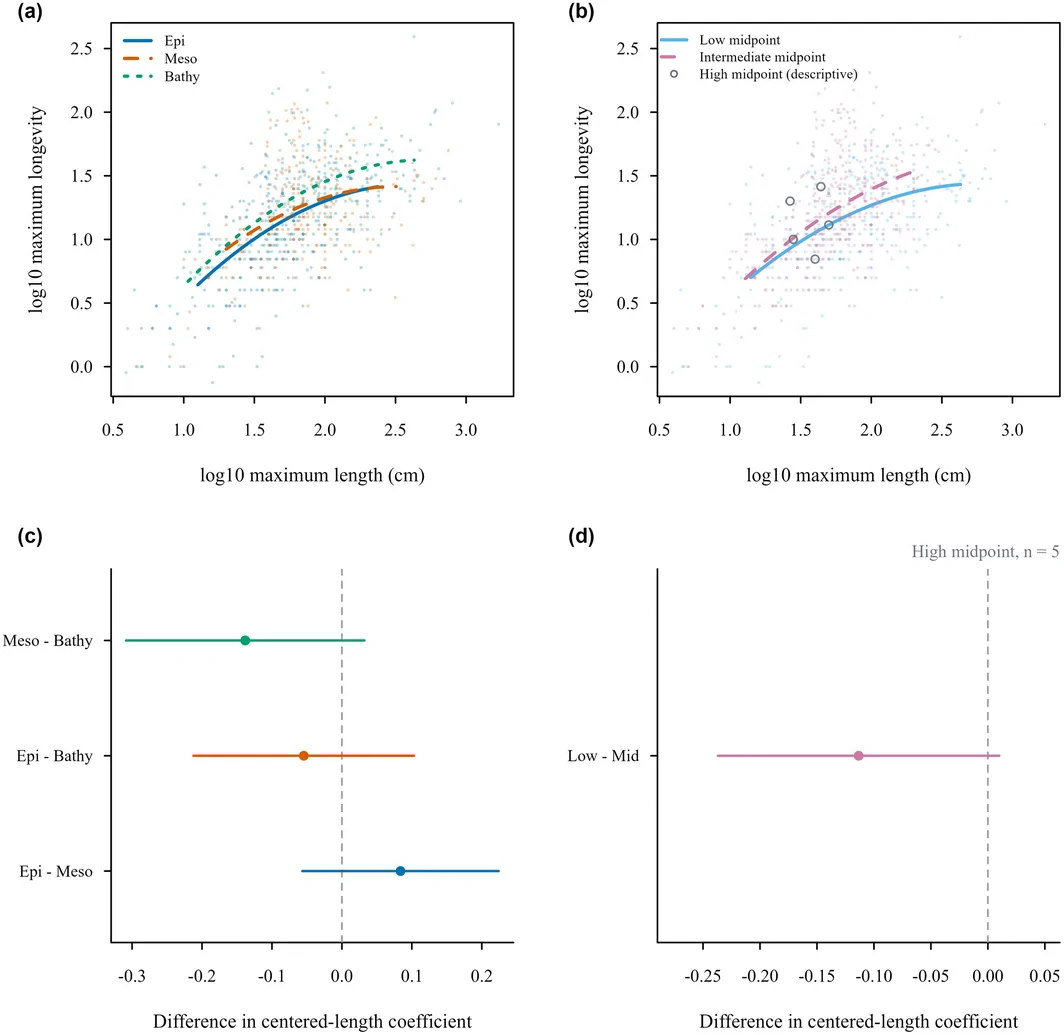
Common‐curvature diagnostic group models. (a) and (b) show observed data and fitted relationships for depth groups and final range‐midpoint latitude groups, with colour and line type identifying groups. (c) shows OLS depth‐group differences in the centred length coefficient. (d) shows the low‐minus‐intermediate midpoint contrast. The high range‐midpoint group contained five species and is shown descriptively; its contrasts were not estimated or interpreted.

**FIGURE 4:**
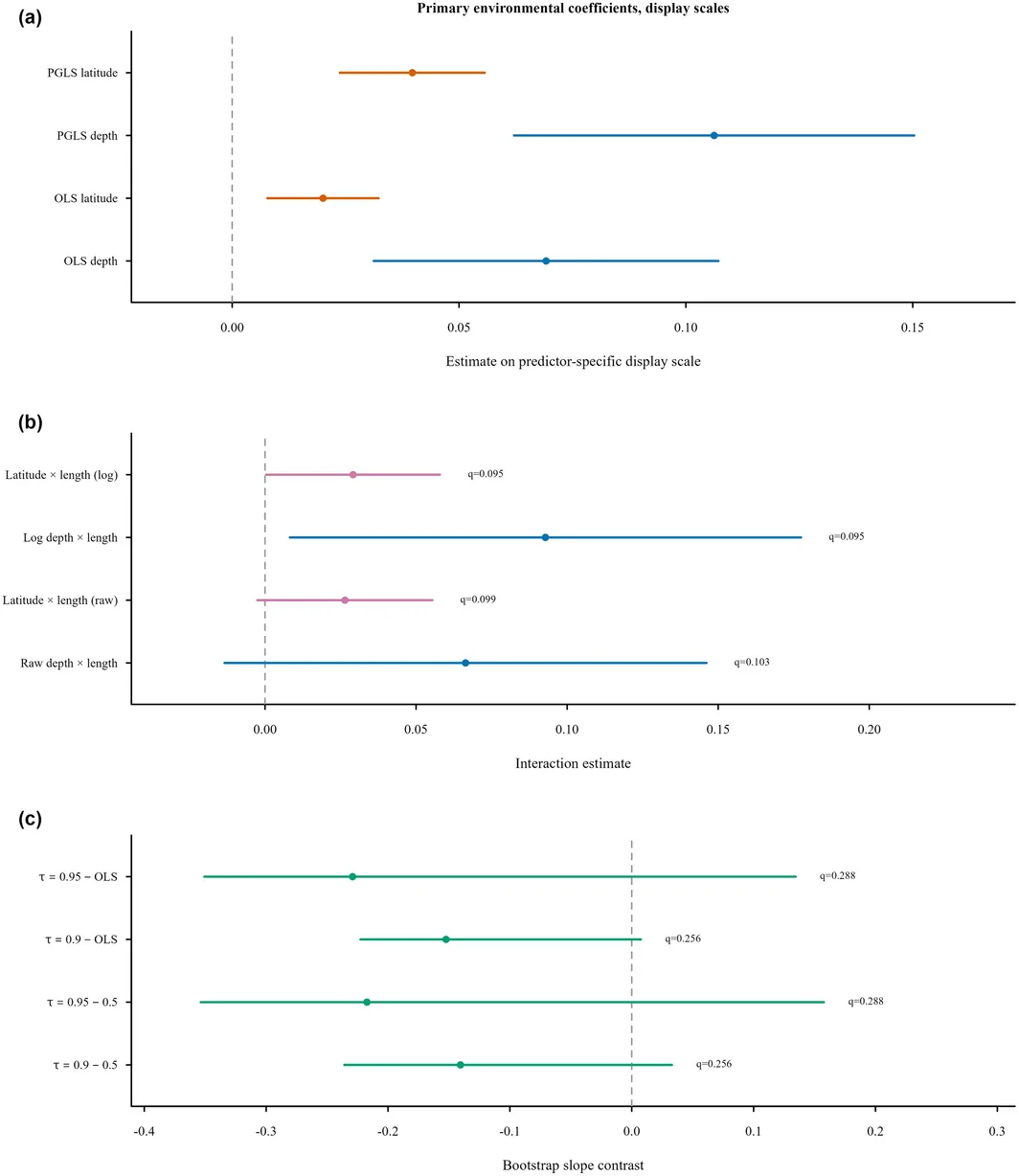
Environmental coefficients and slope diagnostics. (a) shows primary OLS and PGLS depth and latitude coefficients on predictor‐specific display scales; magnitudes are not standardized and should not be compared directly across predictors. (b) shows continuous size‐by‐environment interaction sensitivities with FDR *q*‐values. (c) shows *B* = 5000 linear length‐quantile contrasts. The panels summarize associations and distributional diagnostics, not causal mechanisms.

**TABLE 3:** Diagnostic group, continuous‐interaction and quantile contrasts.

Family	Contrast	Estimate	SE	95% CI	*p*	FDR *q*	Role
OLS depth‐group slopes	Epi—Meso	0.0838	0.0715	−0.0566 to 0.2241	0.242	0.362	Diagnostic
OLS depth‐group slopes	Epi—Bathy	−0.0545	0.0803	−0.2121 to 0.1032	0.498	0.498	Diagnostic
OLS depth‐group slopes	Meso—Bathy	−0.1382	0.0868	−0.3086 to 0.0322	0.112	0.335	Diagnostic
OLS latitude‐group slopes	Low midpoint—Intermediate midpoint	−0.1134	0.0629	−0.2369 to 0.0101	0.072	0.216	Diagnostic; high group descriptive
PGLS depth‐group slopes	Epi—Meso	−0.0309	0.0748	−0.1777 to 0.1160	0.680	0.680	Diagnostic matched subset
PGLS depth‐group slopes	Epi—Bathy	0.0439	0.0989	−0.1503 to 0.2381	0.657	0.680	Diagnostic matched subset
PGLS depth‐group slopes	Meso—Bathy	0.0747	0.0907	−0.1034 to 0.2529	0.410	0.680	Diagnostic matched subset
PGLS latitude‐group slopes	Low midpoint—Intermediate midpoint	0.0722	0.0675	−0.0604 to 0.2048	0.286	0.286	Diagnostic; high group descriptive
Continuous interaction sensitivity	centered log10 length × maximum depth (per 1000 m)	0.0664	0.0407	−0.0134 to 0.1461	0.103	0.103	Supplementary sensitivity
Continuous interaction sensitivity	centered log10 length × absolute signed range‐midpoint latitude (per 10°)	0.0265	0.0148	−0.0025 to 0.0554	0.074	0.099	Supplementary sensitivity
Continuous interaction sensitivity	centered log10 length × log10(maximum depth + 1)	0.0928	0.0432	0.0082 to 0.1774	0.032	0.095	Supplementary sensitivity
Continuous interaction sensitivity	centered log10 length × absolute signed range‐midpoint latitude (per 10°)	0.0291	0.0147	0.0004 to 0.0579	0.048	0.095	Supplementary sensitivity
Length quantile bootstrap	*β*_0.90_ − *β*_0.50_	−0.1406		−0.2358 to 0.0330	0.128	0.256	B = 5000; distributional diagnostic
Length quantile bootstrap	*β*₀.₉₅ − *β*_0.50_	−0.2173		−0.3537 to 0.1577	0.288	0.288	B = 5000; distributional diagnostic
Length quantile bootstrap	*β*_0.90_ − *β*_OLS_	−0.1524		−0.2226 to 0.0075	0.068	0.256	B = 5000; distributional diagnostic
Length quantile bootstrap	*β*₀.₉₅ − *β*_OLS_	−0.2291		−0.3507 to 0.1345	0.249	0.288	B = 5000; distributional diagnostic

*Note*: Epi = epipelagic‐associated; Meso = mesopelagic‐associated; Bathy = bathypelagic‐associated. Contrasts follow the first‐listed minus second‐listed group. *q*‐values use the Benjamini–Hochberg procedure within the defined comparison families. The high range‐midpoint group is descriptive. Quantile intervals are *B* = 5000 species‐bootstrap intervals.

Taxonomic sensitivities retained a positive depth coefficient in Teleostei‐only (0.0832, 95% CI 0.0405 to 0.1260, *p* < 0.001), broad‐Class‐adjusted (0.0720, 95% CI 0.0341 to 0.1100, *p* < 0.001) and Family‐clustered CR2 analyses (0.0692, 95% CI 0.0094 to 0.1289, *p* = 0.026). Latitude remained positive in the first two models, but the Family‐clustered interval included zero (0.0200, 95% CI −0.0197 to 0.0598, *p* = 0.316). The 75‐species elasmobranch‐only model retained a positive depth coefficient, but its smaller within‐class support limits subgroup interpretation (Table S25).

### 3.5 Phylogenetic, quantile and validation diagnostics

Within the 605‐species FishTree‐matched subset, PGLS coefficients were positive for centred length (0.6693, 95% CI 0.5830 to 0.7555), maximum depth (0.1062, 95% CI 0.0621 to 0.1503) and the range‐midpoint latitude proxy (0.0397, 95% CI 0.0237 to 0.0557); all had *p* < 0.001. The quadratic term was −0.1397 (95% CI −0.2723 to −0.0070, *p* = 0.039), Pagel's *λ* was 0.887 and adjusted *R* ^2^ was 0.352. Grouped contrasts were similar when *λ* was fixed at 0.887 or estimated independently, and none met its defined FDR threshold. These results apply only to the taxonomically restricted matched subset (Figure 5; Tables S8, S9 and S24). Complete PGLS group contrasts are reported in Table S12.

**FIGURE 5:**
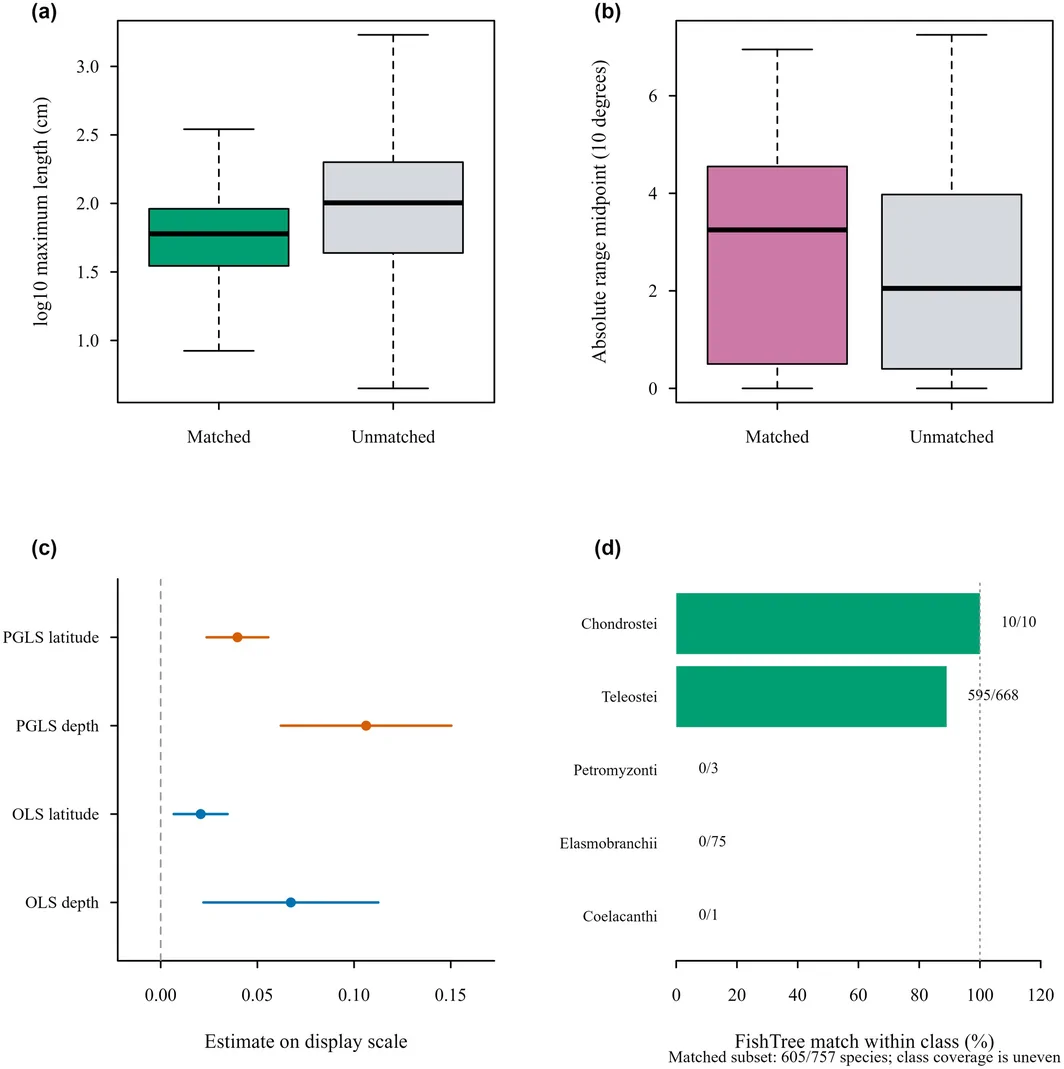
FishTree‐matched robustness and taxonomic coverage. (a and b) compare log10 maximum length and absolute range‐midpoint latitude between matched and unmatched species. (c) compares matched‐subset OLS and PGLS environmental coefficients on predictor‐specific display scales. (d) shows FishTree coverage within each class. The 605‐species subset was dominated by ray‐finned fishes and excluded all canonical elasmobranchs. Pagel's *λ* for the primary environmental model was 0.887; PGLS is a matched‐subset branch‐length covariance sensitivity, not a full‐dataset phylogenetic correction. Length‐ and mass‐based coefficients come from parallel size‐predictor models unless a combined‐size control is explicitly identified.

Linear quantile slopes declined at the highest quantiles, but all B = 5000 length‐contrast intervals included zero. For *β*_0.90_ − *β*_0.50_, the estimate was −0.1406 (95% CI −0.2358 to 0.0330, *p* = 0.128, *q* = 0.256); the quadratic quantile local‐slope intervals also included zero. The quantile analyses therefore did not indicate broad upper‐tail steepening (Figure 6; Tables S13–S16).

**FIGURE 6:**
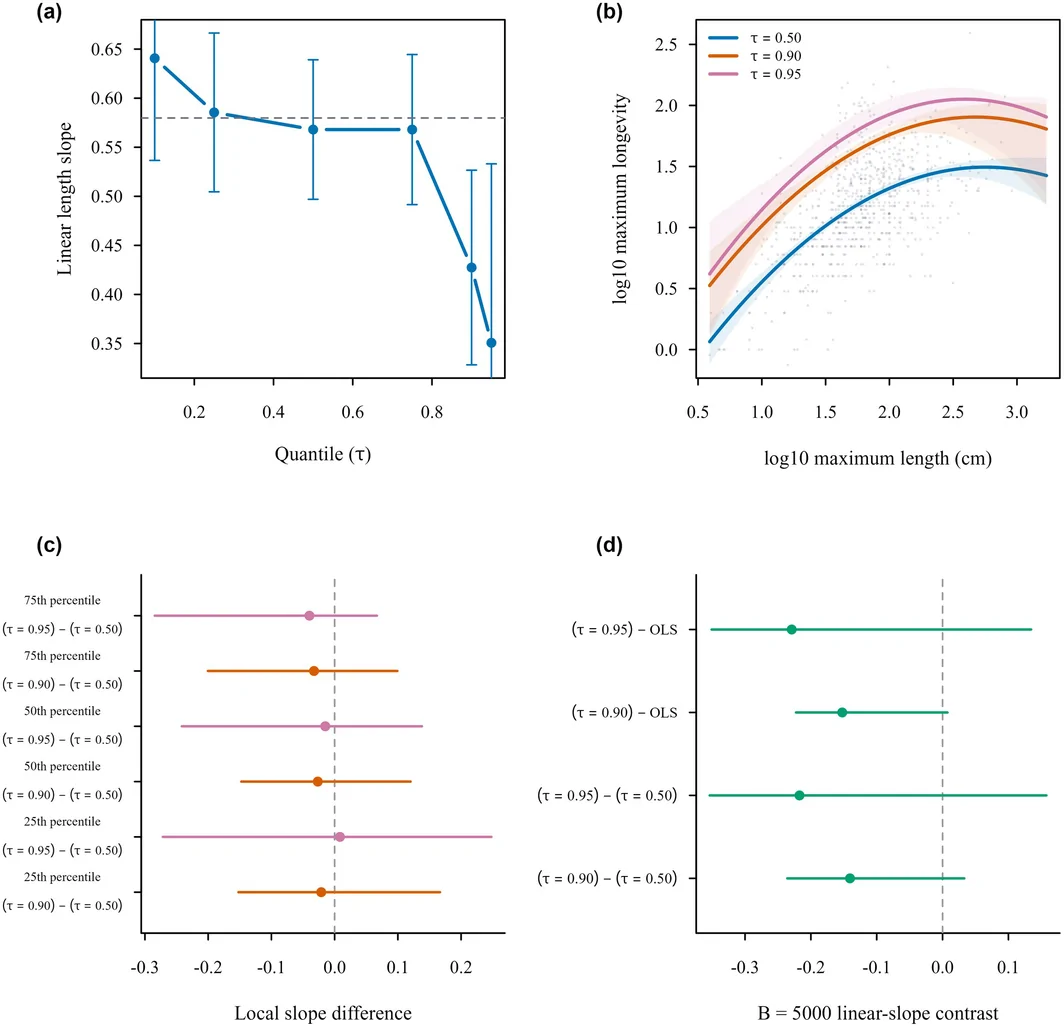
Linear and nonlinear quantile diagnostics. (a) shows linear length slopes across quantiles with uncertainty and the OLS comparator. (b) shows quadratic curves at *τ* = 0.50, *τ* = 0.90 and *τ* = 0.95 with bootstrap bands. Panel c reports local upper‐minus‐median slope differences at the 25th, 50th and 75th percentiles of log10 length, with each *τ* contrast identified directly in the axis labels. (d) shows *B* = 5000 linear slope contrasts. All principal intervals include zero and do not indicate broad upper‐tail steepening.

Predictive *R* ^2^ increased from 0.352 for quadratic length alone to 0.368 after adding maximum depth and to 0.376 after additionally adding the latitude proxy. Under Family blocking, predictive *R* ^2^ increased from 0.316 to 0.321 after adding maximum depth but decreased to 0.273 after additionally adding latitude. Under Order blocking, the corresponding nested sequence was 0.331, 0.342 and 0.277. These internal diagnostics indicate that the latitude term performed less consistently when entire families or orders were withheld; they are not claims of external predictive accuracy (Tables S22 and S23). Species‐random diagnostic summaries are shown in Figure S2, while full validation and reproducibility outputs are provided in Section S6.

## 4 DISCUSSION

### 4.1 Body size and functional form

Body size remained the most consistent correlate of observed maximum longevity, in line with broad life‐history and metabolic expectations. Larger fishes often grow more slowly, mature later and experience lower mass‐specific metabolic rates, while increased size can reduce exposure to some sources of predation and demographic turnover (Brown et al., 2004; Charnov, 1993; Charnov & Gillooly, 2004; Stearns, 1992). These links make body size a useful comparative baseline, but no single allometric exponent is likely to describe every lineage or every part of the size range. Ageing trajectories vary widely across vertebrates, and fishes show substantial departures from simple size‐based expectations even among broadly comparable taxa (Jones et al., 2014; Stark, 2022).

The positive size–longevity association was not linear across the observed size range. The quadratic model indicated broad curvature: longevity increased strongly through much of the observed size range, but the incremental association weakened among larger species. The flexible GAM sensitivity showed a similar broad shape but did not resolve the extreme tail. The traits accompanying greater size—growth rate, maturation schedule, reproductive allocation, and mortality exposure—do not change in fixed proportion across all fishes. A curved relationship is biologically plausible and is consistent with the reduction in systematic residual structure relative to the linear model. The quadratic provides a compact approximation to broad attenuation; it does not establish a universal polynomial law or a biological downturn among the largest fishes.

That uncertainty is especially relevant for the largest and longest‐lived fishes. Only 15 species occurred beyond the fitted zero‐derivative location; several were influential observations, and this part of the curve lay outside the central common‐support window. The location is not interpreted as a biological threshold, and the descending tail is not evidence that maximum longevity declines among the largest fishes. Exceptional cases such as the Greenland shark *Somniosus microcephalus* and long‐lived Pacific rockfishes *Sebastes* spp. show how lineage‐specific growth, maturation and ageing strategies can generate longevity far beyond a simple size prediction (Kolora et al., 2021; Nielsen et al., 2016). The data support broad attenuation at large body sizes more strongly than any exact turning point or decline.

Mass‐based sensitivities produced the same general positive size association, which increases confidence that the result is not unique to the selected length measure. Their inferential value remains secondary because completed mass was partly reconstructed and strongly collinear with length. The analysis therefore does not support a single scaling exponent across all fishes. Body size remains a central comparative axis, but its association with maximum longevity changes across the observed size range, while residual variation may reflect lineage‐specific energetic trade‐offs, ecological conditions, extrinsic mortality and differences in sampling effort (Stark, 2022; Stark et al., 2018, 2020). Parallel mass‐based group sensitivities are shown in Figure S3.

### 4.2 Maximum depth beyond body size

Maximum depth was the environmental variable that showed the greatest consistency across the analytical framework. Its positive coefficient was retained in the full model, central common‐support subsets, Teleostei‐only and broad‐Class‐adjusted models and under Family‐clustered uncertainty. The FishTree‐matched PGLS model produced the same direction, while a supplementary analysis of elasmobranchs indicated that the pattern was not restricted to ray‐finned fishes. The positive depth association was robust to these alternative specifications and taxonomic sensitivity analyses. This consistency does not identify a causal process or rule out residual confounding.

The result is broadly consistent with the slow life histories documented in many deep‐living fishes. Deep‐sea taxa frequently combine slow growth, delayed maturation, low natural mortality and extended lifespan, although these traits vary continuously rather than forming a single deep‐sea syndrome (Drazen & Haedrich, 2012; Priede & Froese, 2013). Cooler environments may reduce metabolic turnover and alter growth–mortality relationships, while low resource availability can favour conservative energy allocation and prolonged development (Black et al., 2021; Gislason et al., 2010; Pauly, 1980). Oxygen availability may further modify the temperature and body‐size dependence of metabolism in aquatic ectotherms (Rubalcaba et al., 2020). These processes are not mutually exclusive, and their relative importance probably differs among lineages and habitats.

Variation in extrinsic mortality offers an additional route linking depth to longevity. Predation regimes, physical disturbance and fishing exposure can all change with depth. Life‐history theory predicts that sustained differences in external mortality may shape growth, maturation, and investment in somatic maintenance, although the direction of this relationship depends on ecological context and cannot be inferred from a single range variable (Dańko et al., 2018; de Vries et al., 2023). Some deep‐associated species may experience lower exploitation or predation, whereas others remain exposed during shallower life stages or through deep‐water fisheries. Maximum depth alone cannot distinguish these histories.

Depth entered the models mainly as an additive term. Once the common curvature of the size–longevity relationship was retained, neither grouped nor continuous interaction models showed a stable depth‐related change in the centred length coefficient. Species reaching greater depths therefore tended to have higher expected longevity across the observed size range, with little evidence that depth systematically altered the strength of the size association itself. This distinction revises the stronger environmental‐modulation interpretation considered earlier in the study. Maximum depth appears to summarize a broad ecological and life‐history context that adds information beyond body size, while the underlying pathways remain unresolved.

### 4.3 Why group‐specific slopes are weak

No stable group‐specific scaling differences were detected. Separate fitted lines can appear to diverge when environmental groups occupy different parts of a curved relationship. In the present dataset, larger species were not distributed evenly among depth and latitude categories, and the high range‐midpoint group contained only five species. A linear fit within each category would combine genuine between‐group differences with the local slope of the shared nonlinear relationship. Retaining a common quadratic term removed much of this ambiguity. The remaining differences in the centred length coefficient were small relative to their uncertainty, and none of the contrasts met the defined FDR threshold of *q* < 0.05.

The grouped analysis also shows what is lost when continuous gradients are divided into broad classes. Categories are useful for displaying coverage and identifying poorly represented combinations, but their boundaries are analytical conventions. Species close to a depth or latitude threshold may be ecologically similar while being assigned to different groups. At the same time, sparse cells can exert disproportionate influence on an estimated line. The overlap plots, occupancy counts and common‐support analyses were therefore central to interpretation, not simply supplementary diagnostics. They showed that all major depth groups contained species across broad length intervals, but depth and latitude were not fully crossed and the extreme latitude category lacked enough observations for formal inference. Restricting the analysis to supported regions retained the additive depth association but did not reveal consistent slope differences.

The quantile results point in the same direction. Upper edges in ecological scatterplots can look biologically meaningful even when they reflect unequal sample density, measurement error or the changing variance of the response (Cade & Noon, 2003; Scharf et al., 1998). Here, the estimated slopes at *τ* = 0.90 and *τ* = 0.95 were not steeper than the median or OLS slopes, and the nonlinear local‐slope contrast intervals also included zero. The data consequently do not support a distinct upper boundary that becomes more size‐dependent under particular environmental conditions.

Together, the interaction and quantile results support a narrower interpretation in which environmental context is associated with differences in expected longevity around a shared, curved size relationship. The grouped fits remain useful descriptions of the observed sample, but they should not be treated as separate ecological regimes. More decisive tests of slope variation will require denser sampling across the joint size–depth–latitude space, especially among very large species and at the highest range‐midpoint latitudes.

### 4.4 Latitude and phylogenetic uncertainty

Latitude produced a less coherent pattern than maximum depth. The absolute signed range midpoint was positively associated with longevity in the primary OLS model and in the FishTree‐matched PGLS analysis, but that result depended on how geographic range was summarized. Coefficients based on the legacy endpoint mean and maximum absolute endpoint were uncertain; the strict common‐support interval included zero, and the latitude term performed less consistently when entire families or orders were withheld. Its interval also included zero under Family‐clustered uncertainty. These differences indicate that the significant primary coefficient captures information specific to one range‐based representation; it does not provide a stable estimate of a general latitude effect.

A species' range midpoint is only a coarse approximation to environmental exposure. Nearly 40% of the species in the dataset crossed the equator, and two species with similar midpoint values can occupy very different combinations of hemispheres, seasonal regimes and latitudinal range widths. Ocean currents, regional productivity and the vertical thermal structure of the water column can also decouple latitude from temperature. Earlier evidence for latitudinal variation in lifespan has often concerned within‐species populations or more direct geographical comparisons (Munch & Salinas, 2009). The present analysis instead compares range limits across distantly related species. The two settings address different biological questions, and the current proxy cannot distinguish thermal exposure from biogeographic history or range geometry.

Phylogenetic modelling does not remove this measurement limitation. The positive PGLS coefficient describes covariance within the 605‐species FishTree subset, which was dominated by Teleostei and excluded every elasmobranch in the canonical dataset. Similar grouped contrasts under fixed and independently estimated Pagel's *λ* show that those particular results were not sensitive to one *λ* setting. They do not compensate for missing lineages or an imperfect environmental proxy. Comparative methods can control covariance among represented species, but their inferential reach remains tied to tree coverage, trait measurement and the composition of the matched sample (Felsenstein, 1985; Freckleton et al., 2002; Symonds & Blomberg, 2014). Latitude should therefore remain an exploratory result. A stronger test will require occurrence‐weighted geographical and thermal exposure, broader phylogenetic coverage and explicit separation of within‐lineage from among‐lineage variation.

### 4.5 Limitations and future work

Some uncertainty enters before any model is fitted. Maximum longevity is a record statistic whose value increases with sampling effort and depends on ageing method, validation quality and the availability of exceptionally old individuals. These issues are especially consequential for long‐lived fishes, in which small ageing biases can translate into large errors in estimated lifespan (Campana, 2001). Maximum length is also heterogeneous because the database combines total, standard and fork length, while completed mass includes reconstructed values. The TL‐only and mass sensitivities help to assess these choices, but they cannot remove measurement error from the original records.

Selection and ascertainment may introduce a second layer of uncertainty. The complete‐case dataset necessarily favours species for which longevity, length, depth and latitude have all been documented. Well‐studied, large‐bodied, commercially important or unusually long‐lived taxa may therefore be overrepresented. Maximum longevity, maximum length and maximum recorded depth are all extreme values, and each can increase as research effort accumulates. Shared ascertainment could strengthen their observed associations even when the underlying biological relationships are weaker. The current data contain no consistent species‐level measure of study effort with which to model this process directly.

The environmental variables create a third limitation. Neither variable resolves ontogenetic habitat shifts, seasonal movement, oxygen exposure, productivity, fishing mortality, or local predation. Future work should link validated population‐level ages to occurrence‐weighted depth and temperature histories, exploitation records and local mortality schedules. Hierarchical models could then separate variation among populations, species, and lineages, while measurement‐error models could propagate uncertainty in age, body size and environmental exposure. Such data would move the analysis from broad macroecological pattern towards direct tests of demographic and physiological hypotheses.

## 5 CONCLUSION

Maximum longevity in marine‐occurring fishes was positively but nonlinearly related to body size, with a parsimonious quadratic model capturing broad attenuation across the observed range. Maximum depth showed the most consistent positive environmental association beyond size. Latitude varied among range‐based metrics, common‐support restrictions, clustered uncertainty and taxonomically blocked diagnostics, so it remains exploratory. Group‐specific slope contrasts and continuous interactions did not indicate robust environmental variation in the centred length coefficient after FDR adjustment, and quantile analyses did not indicate broad upper‐tail steepening. These results describe bounded macroecological associations, not tested causal mechanisms.

## AUTHOR CONTRIBUTIONS

L.L.: Conceptualization, Methodology, Data curation, Formal analysis, Visualization, Writing – original draft, Writing – review and editing. Z.X.: Conceptualization, Methodology, Validation, Writing – review and editing.

## FUNDING INFORMATION

The authors have nothing to report.

## CONFLICT OF INTEREST STATEMENT

The authors declare no competing interests.

## Supporting information


**Figure S1.** Audited FishBase filtering flow in executable code order. Counts are species retained after each successive filter, with stage‐specific exclusions shown at right. The archived species table was already unique by SpecCode. Repeated stock records were deterministically summarized within SpecCode before a one‐to‐one join; signed northern and southern limits were the maximum and minimum reconstructed endpoints, respectively, and the final fail‐fast check confirmed zero duplicate SpecCode values. The canonical dataset contains 757 species; 605 matched FishTree and 152 unmatched species were documented without grafting.
**Figure S2**. Fixed‐seed, SpecCode‐randomized five‐fold diagnostic metrics for the linear comparator, retained quadratic model, environmental additions and combined‐size sensitivity. These are internal model checks; complete fold maps and fold‐level metrics are archived.
**Figure S3**. Mass‐based group sensitivities. Mass relationships are shown separately from primary length models because completed mass was partly derived.
**Figure S4**. Collinearity and body‐mass provenance. Length and mass have high variance inflation factors; depth and latitude do not. Provenance labels match Table 1.
**Figure S5**. Pairwise predictor‐overlap and common‐support diagnostics. Pairwise scatterplots, group‐wise length distributions and cell counts show incomplete depth‐by‐latitude coverage; the high midpoint group is descriptive.
**Table S1**. Variable dictionary and transformations.
**Table S2**. Weight‐provenance summary.
**Table S3**. Maximum‐length type inventory and TL‐only sensitivity.
**Table S4**. FishTree matching, taxonomic coverage and unmatched species.
**Table S5**. FishTree chronogram diagnostics.
**Table S6**. Functional‐form comparison, decision and upper‐tail support.
**Table S7**. Selected OLS outputs.
**Table S8**. Selected PGLS outputs.
**Table S9**. Matched‐subset OLS‐PGLS comparison.
**Table S10**. Quadratic environmental models.
**Table S11**. Common‐curvature OLS group‐slope contrasts.
**Table S12**. PGLS common‐curvature group contrasts.
**Table S13**. Linear quantile slope estimates.
**Table S14**. Linear quantile bootstrap contrasts and upper‐tail support.
**Table S15**. Quadratic quantile coefficients and local slope contrasts.
**Table S16**. Quadratic quantile curve predictions.
**Table S17**. Collinearity diagnostics.
**Table S18**. Predictor overlap and length‐bin occupancy.
**Table S19**. Continuous‐interaction sensitivity.
**Table S20**. Common‐support and trimmed sensitivities.
**Table S21**. Latitude reconstruction and evidence hierarchy.
**Table S22**. Fixed‐seed species‐random diagnostic validation.
**Table S23**. Taxonomy‐blocked diagnostic validation.
**Table S24**. Grouped PGLS λ sensitivity.
**Table S25**. Taxonomic‐composition and Family‐clustered sensitivity.
**Section S1**. Dataset assembly, provenance and variable definitions.
**Section S2**. FishTree matching and chronogram diagnostics.
**Section S3**. Functional form and environmental model outputs.
**Section S4**. Group‐slope and quantile diagnostics.
**Section S5**. Common support and sensitivity analyses
**Section S6**. Diagnostic validation and reproducibility.

## Data Availability

Data and code supporting this study are available from Zenodo at https://doi.org/10.5281/zenodo.21769695. The reproducibility archive contains the canonical data, cached source queries, FishTree files, scripts, model outputs, figures, tables, session information and strict check/rebuild instructions.

## References

[jfb70629-bib-0001] Andersen, K. H. , Berge, T. , Gonçalves, R. J. , Hartvig, M. , Heuschele, J. , Hylander, S. , Jacobsen, N. S. , Lindemann, C. , Martens, E. A. , Neuheimer, A. B. , Olsson, K. , Palacz, A. , Prowe, A. E. , Sainmont, J. , Traving, S. J. , Visser, A. W. , Wadhwa, N. , & Kiørboe, T. (2016). Characteristic sizes of life in the oceans, from bacteria to whales. Annual Review of Marine Science, 8, 217–241. 10.1146/annurev-marine-122414-034144 26163011

[jfb70629-bib-0002] Benjamini, Y. , & Hochberg, Y. (1995). Controlling the false discovery rate: A practical and powerful approach to multiple testing. Journal of the Royal Statistical Society. Series B, Statistical Methodology, 57, 289–300. 10.1111/j.2517-6161.1995.tb02031.x

[jfb70629-bib-0003] Black, B. A. , Drazen, J. C. , Grote, B. , Koslow, J. A. , Andrews, A. H. , Cailliet, G. M. , & Roark, E. B. (2021). Environmental, evolutionary, and ecological drivers of slow life histories in deep‐sea fishes. Marine Ecology Progress Series, 658, 1–26. 10.3354/meps13591

[jfb70629-bib-0004] Boettiger, C. , Temple Lang, D. , & Wainwright, P. C. (2012). Rfishbase: Exploring, manipulating and visualizing FishBase data from R. Journal of Fish Biology, 81, 2030–2039. 10.1111/j.1095-8649.2012.03464.x 23130696

[jfb70629-bib-0005] Brown, J. H. , Gillooly, J. F. , Allen, A. P. , Savage, V. M. , & West, G. B. (2004). Toward a metabolic theory of ecology. Ecology, 85, 1771–1789. 10.1890/03-9000

[jfb70629-bib-0006] Cade, B. S. , & Noon, B. R. (2003). A gentle introduction to quantile regression for ecologists. Frontiers in Ecology and the Environment, 1, 412–420. 10.1890/1540-9295(2003)001[0412:AGITQR]2.0.CO;2

[jfb70629-bib-0007] Campana, S. E. (2001). Accuracy, precision and quality control in age determination, including a review of the use and abuse of age validation methods. Journal of Fish Biology, 59, 197–242. 10.1111/j.1095-8649.2001.tb00127.x

[jfb70629-bib-0008] Chang, J. , Rabosky, D. L. , Smith, S. A. , & Alfaro, M. E. (2019). An R package and online resource for macroevolutionary studies using the ray‐finned fish tree of life. Methods in Ecology and Evolution, 10, 1118–1124. 10.1111/2041-210X.13182

[jfb70629-bib-0009] Charnov, E. L. (1993). Life history invariants: Some explorations of symmetry in evolutionary ecology. Oxford University Press.

[jfb70629-bib-0010] Charnov, E. L. , & Gillooly, J. F. (2004). Size and temperature in the evolution of fish life histories. Integrative and Comparative Biology, 44, 494–497. 10.1093/icb/44.6.494 21676735

[jfb70629-bib-0011] Dańko, M. J. , Burger, O. , Argasiński, K. , & Kozłowski, J. (2018). Extrinsic mortality can shape life‐history traits, including senescence. Evolutionary Biology, 45, 395–404. 10.1007/s11692-018-9458-7 PMC6223763 30459480

[jfb70629-bib-0012] de Vries, C. , Galipaud, M. , & Kokko, H. (2023). Extrinsic mortality and senescence: A guide for the perplexed. Peer Community Journal, 3, e29. 10.24072/pcjournal.253

[jfb70629-bib-0013] Dormann, C. F. , Elith, J. , Bacher, S. , Buchmann, C. , Carl, G. , Carre, G. , Garcia Marquez, J. R. , Gruber, B. , Lafourcade, B. , Leitao, P. J. , Muenkemueller, T. , McClean, C. , Osborne, P. E. , Reineking, B. , Schroeder, B. , Skidmore, A. K. , Zurell, D. , & Lautenbach, S. (2013). Collinearity: A review of methods to deal with it and a simulation study evaluating their performance. Ecography, 36, 27–46. 10.1111/j.1600-0587.2012.07348.x

[jfb70629-bib-0014] Drazen, J. C. , & Haedrich, R. L. (2012). A continuum of life histories in deep‐sea demersal fishes. Deep‐Sea Research Part I, 61, 34–42. 10.1016/j.dsr.2011.11.002

[jfb70629-bib-0015] Felsenstein, J. (1985). Phylogenies and the comparative method. The American Naturalist, 125, 1–15. 10.1086/284325 31094602

[jfb70629-bib-0016] Freckleton, R. P. , Harvey, P. H. , & Pagel, M. (2002). Phylogenetic analysis and comparative data: A test and review of evidence. The American Naturalist, 160, 712–726. 10.1086/343873 18707460

[jfb70629-bib-0017] Froese, R. , & Pauly, D. (2026). FishBase. World Wide Web electronic publication. www.fishbase.org (accessed 10 June 2026).

[jfb70629-bib-0018] Gislason, H. , Daan, N. , Rice, J. C. , & Pope, J. G. (2010). Size, growth, temperature and the natural mortality of marine fish. Fish and Fisheries, 11, 149–158. 10.1111/j.1467-2979.2009.00350.x

[jfb70629-bib-0019] Jones, O. R. , Scheuerlein, A. , Salguero‐Gómez, R. , Camarda, C. G. , Schaible, R. , Casper, B. B. , Dahlgren, J. P. , Ehrlén, J. , García, M. B. , Menges, E. S. , Quintana‐Ascencio, P. F. , Caswell, H. , Baudisch, A. , & Vaupel, J. W. (2014). Diversity of ageing across the tree of life. Nature, 505, 169–173. 10.1038/nature12789 PMC4157354 24317695

[jfb70629-bib-0020] Koenker, R. (2005). Quantile Regression. Cambridge University Press. 10.1017/CBO9780511754098

[jfb70629-bib-0021] Koenker, R. , & Bassett, G. (1978). Regression quantiles. Econometrica, 46, 33–50. 10.2307/1913643

[jfb70629-bib-0022] Kolora, S. R. R. , Owens, G. L. , Vazquez, J. M. , Stubbs, A. , Chatla, K. , Jainese, C. , Seeto, K. , McCrea, M. , Sandel, M. W. , Vianna, J. A. , Maslenikov, K. , Bachtrog, D. , Orr, J. W. , Love, M. , & Sudmant, P. H. (2021). Origins and evolution of extreme life span in Pacific Ocean rockfishes. Science, 374, 842–847. 10.1126/science.abg5332 PMC8923369 34762458

[jfb70629-bib-0023] Munch, S. B. , & Salinas, S. (2009). Latitudinal variation in lifespan within species is explained by the metabolic theory of ecology. Proceedings of the National Academy of Sciences, 106, 13860–13864. 10.1073/pnas.0900300106 PMC2728985 19666552

[jfb70629-bib-0024] Nielsen, J. , Hedeholm, R. B. , Heinemeier, J. , Bushnell, P. G. , Christiansen, J. S. , Olsen, J. , Ramsey, C. B. , Brill, R. W. , Simon, M. , Steffensen, K. F. , & Steffensen, J. F. (2016). Eye lens radiocarbon reveals centuries of longevity in the Greenland shark (*Somniosus microcephalus*). Science, 353, 702–704. 10.1126/science.aaf1703 27516602

[jfb70629-bib-0025] Orme, D. , Freckleton, R. , Thomas, G. , Petzoldt, T. , Fritz, S. , Isaac, N. , & Pearse, W. (2025). caper: Comparative analyses of phylogenetics and evolution in R. R package version 1.0.4. 10.32614/CRAN.package.caper

[jfb70629-bib-0026] Paradis, E. , & Schliep, K. (2019). Ape 5.0: An environment for modern phylogenetics and evolutionary analyses in R. Bioinformatics, 35, 526–528. 10.1093/bioinformatics/bty633 30016406

[jfb70629-bib-0027] Pauly, D. (1980). On the interrelationships between natural mortality, growth parameters, and mean environmental temperature in 175 fish stocks. ICES Journal of Marine Science, 39, 175–192. 10.1093/icesjms/39.2.175

[jfb70629-bib-0028] Priede, I. G. , & Froese, R. (2013). Colonization of the deep sea by fishes: Evidence from seamounts. Journal of Fish Biology, 83, 1528–1550. 10.1111/jfb.12265 PMC4291108 24298950

[jfb70629-bib-0029] R Core Team . (2026). R: A language and environment for statistical computing. R Foundation for Statistical Computing. 10.32614/R.manuals

[jfb70629-bib-0030] Rabosky, D. L. , Chang, J. , Title, P. O. , Cowman, P. F. , Sallan, L. , Friedman, M. , Kaschner, K. , Garilao, C. , Near, T. J. , Coll, M. , & Alfaro, M. E. (2018). An inverse latitudinal gradient in speciation rates of marine fishes. Nature, 559, 392–395. 10.1038/s41586-018-0273-1 29973726

[jfb70629-bib-0031] Rubalcaba, J. G. , Verberk, W. C. E. P. , Hendriks, A. J. , Saris, H. J. , & Olalla‐Tárraga, M. A. (2020). Oxygen limitation may affect the temperature and size dependence of metabolism in aquatic ectotherms. Proceedings of the National Academy of Sciences of the United States of America, 117, 31963–31968. 10.1073/pnas.2003292117 PMC7749359 33257544

[jfb70629-bib-0032] Scharf, F. S. , Juanes, F. , & Sutherland, M. (1998). Inferring ecological relationships from the edges of scatter diagrams: Comparison of regression techniques. Ecology, 79, 448–460. 10.1890/0012-9658(1998)079[0448:IERFTE]2.0.CO;2

[jfb70629-bib-0033] Stark, G. (2022). Large and expensive brain comes with a short lifespan: The relationship between brain size and longevity among fish taxa. Journal of Fish Biology, 101, 92–99. 10.1111/jfb.15074 PMC9544989 35482011

[jfb70629-bib-0034] Stark, G. , Pincheira‐Donoso, D. , & Meiri, S. (2020). No evidence for the ‘rate‐of‐living’ theory across the tetrapod tree of life. Global Ecology and Biogeography, 29, 857–884. 10.1111/geb.13069

[jfb70629-bib-0035] Stark, G. , Tamar, K. , Itescu, Y. , Feldman, A. , & Meiri, S. (2018). Cold and isolated ectotherms: Drivers of reptilian longevity. Biological Journal of the Linnean Society, 125, 730–740. 10.1093/biolinnean/bly153

[jfb70629-bib-0036] Stearns, S. C. (1992). The evolution of life histories. Oxford University Press.

[jfb70629-bib-0037] Symonds, M. R. E. , & Blomberg, S. P. (2014). A primer on phylogenetic generalised least squares. In L. Z. Garamszegi (Ed.), Modern phylogenetic comparative methods and their application in evolutionary biology (pp. 105–130). Springer. 10.1007/978-3-662-43550-2_5

[jfb70629-bib-0038] Wood, S. N. (2017). Generalized additive models: An introduction with R (2nd ed.). Chapman and Hall/CRC. 10.1201/9781315370279

[jfb70629-bib-0039] Zuur, A. F. , Ieno, E. N. , & Elphick, C. S. (2010). A protocol for data exploration to avoid common statistical problems. Methods in Ecology and Evolution, 1, 3–14. 10.1111/j.2041-210X.2009.00001.x

