# Supplementary material for: Body size, functional form and environmental correlates of maximum longevity in marine‐occurring fishes
Source: J Fish Biol. 2026 Aug 30;109(4):2458–74. doi: 10.1111/jfb.70629 (PMC13675504; doi:10.1111/jfb.70629)
Supplement: Supplementary file 1 [file JFB-109-2458-s001.pdf]

Supporting Information

Journal of Fish Biology manuscript 4855323

Body size, functional form and environmental correlates of maximum longevity in marine-occurring fishes

Longyu Li and Zenghui Xu

Complete machine-readable Supplementary Tables S1-S25 are supplied in JFB4855323_Supplementary_Tables_S1-S25.xlsx.

Five non-duplicative supplementary figures are provided in this document and as separate publication-quality files.

Contents

Section S1. Dataset assembly, provenance and variable definitions 3
Section S2. FishTree matching and chronogram diagnostics 7
Section S3. Functional form and environmental model outputs 9
Section S4. Group-slope and quantile diagnostics 15
Section S5. Common support and sensitivity analyses 22
Section S6. Diagnostic validation and reproducibility 32
Supplementary Figure S1 3
Supplementary Figure S2 15
Supplementary Figure S3 15
Supplementary Figure S4 22
Supplementary Figure S5 23
Supplementary Table S1 4
Supplementary Table S2 5
Supplementary Table S3 6
Supplementary Table S4 7
Supplementary Table S5 8
Supplementary Table S6 9
Supplementary Table S7 10
Supplementary Table S8 11
Supplementary Table S9 12
Supplementary Table S10 13
Supplementary Table S11 16
Supplementary Table S12 16
Supplementary Table S13 17
Supplementary Table S14 18
Supplementary Table S15 20
Supplementary Table S16 21
Supplementary Table S17 23
Supplementary Table S18 24
Supplementary Table S19 25
Supplementary Table S20 26
Supplementary Table S21 28
Supplementary Table S22 32
Supplementary Table S23 33
Supplementary Table S24 36
Supplementary Table S25 38

Section S1. Dataset assembly, provenance and variable definitions

This section documents the exact code-order filtering flow, SpecCode handling, analysis-set construction, units, maximum-length types and completed-mass provenance. Figure S1 reports every retained and excluded species count. The FishBase web resource and species-name query were accessed on 10 June 2026; signed stock endpoints and maximum-length-type metadata were queried or refreshed on 13 July 2026. Length-based models do not depend on completed Weight_g provenance.

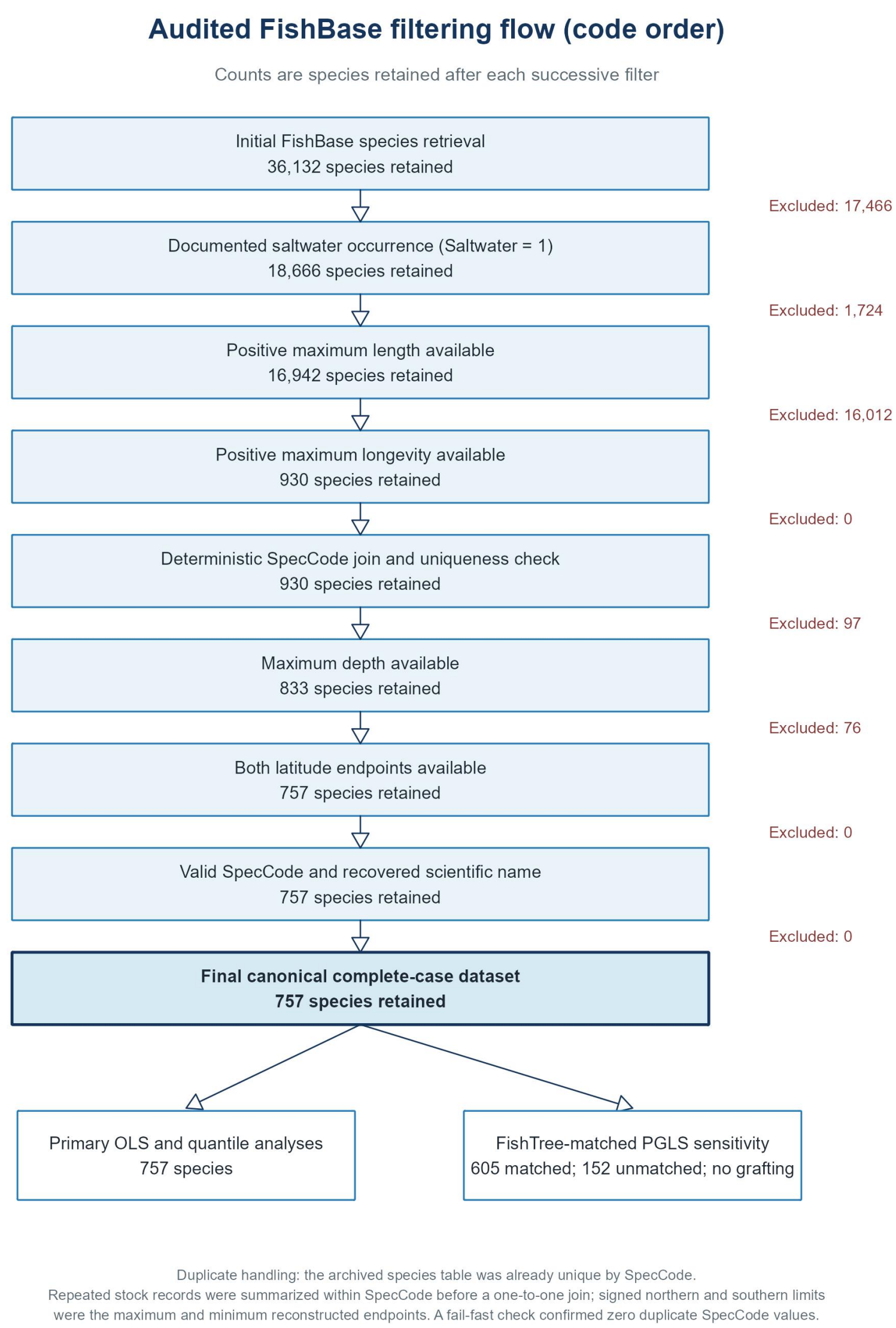

Supplementary Figure S1. Audited FishBase filtering flow in executable code order. Counts are species retained after each successive filter, with stage-specific exclusions shown at right. The archived species table was already unique by SpecCode. Repeated stock records were deterministically summarized within SpecCode before a one-to-one join; signed northern and southern limits were the maximum and minimum reconstructed endpoints, respectively, and the final fail-fast check confirmed zero duplicate SpecCode values. The canonical dataset contains 757 species; 605 matched FishTree and 152 unmatched species were documented without grafting.

Supplementary Table S1. Variable dictionary and transformations.

variable	publication label	definition	transformation or note
SpecCode	FishBase SpecCode	FishBase species identifier.	Stable identifier retained from FishBase.
FBname	Common name	FishBase common name where available.	Missing values indicate that no common name was available.
Species	Scientific name	Recovered scientific name used for FishTree matching and taxonomic reporting.	Not used as a model predictor.
Genus	Genus	Taxonomic genus associated with the scientific name.	Merged from the archived taxonomy matching file.
Family	Family	Taxonomic family associated with the scientific name.	Used for taxonomy-blocked diagnostic folds.
Order	Order	Taxonomic order associated with the scientific name.	Used for taxonomy-blocked diagnostic folds.
Class	Class	Taxonomic class associated with the scientific name.	Taxonomic metadata.
Length	Maximum length	Maximum recorded length used as the primary body-size metric (cm).	log10 transformed; source length type is retained separately.
Weight	Raw body mass	Raw FishBase body mass field (g).	Missing raw values were not used to define the primary length analyses.
LongevityWild	Maximum longevity	Maximum recorded longevity in the wild (years).	Response variable; log10 transformed.
DepthRangeDeep	Maximum depth	Maximum recorded depth (m).	Primary continuous environmental predictor; displayed per 1000 m.
Saltwater	Saltwater flag	FishBase-derived indicator of marine occurrence.	Saltwater = 1 was required during curation.
a_mean	Length-weight coefficient a	Arithmetic mean of available FishBase coefficient a records within SpecCode.	Applied as W = aL^b for documented species-level derivations.
b_mean	Length-weight exponent b	Arithmetic mean of available FishBase exponent b records within SpecCode.	Applied as W = aL^b for documented species-level derivations.
Northernmost	Legacy northern endpoint field	Legacy curated latitude endpoint retained for provenance.	Do not use for final latitude inference; signed endpoints were reconstructed separately.
Southermost	Legacy southern endpoint field	Legacy source field retained under its original column name.	Do not use for final latitude inference; signed endpoints were reconstructed separately.
Weight_g	Completed body mass	Completed body mass field (g).	Raw for 424 species, derived with species-level mean coefficients for 261, and generated by the archived W = 0.01L^3 fallback for 72 entries classified as unresolved in provenance; mass models are sensitivity evidence.
MeanLatitude_deg	Legacy latitude proxy	Legacy latitude field retained for reproducibility.	Not the final latitude metric.
log_Length	log10 maximum length	log10-transformed maximum length (cm).	Primary size predictor before centering.
log_Weight	log10 completed body mass	log10-transformed Weight_g.	Mass sensitivity predictor.
log_Longevity	log10 maximum longevity	log10-transformed maximum longevity (years).	Model response.
DepthGroup	Depth group	Diagnostic stratum defined from maximum depth.	Epi <200 m; Meso 200–<1000 m; Bathy ≥1000 m.
MeanAbsoluteRangeLimitLatitude_deg	Legacy absolute-endpoint mean proxy	Mean magnitude of the legacy northern and southern endpoint fields (degrees).	Sensitivity metric; not a distribution centroid.
LegacyLatitudeGroup	Legacy latitude category	Archived category based on the legacy latitude proxy.	Retained for audit only; not used in final grouped models.
LengthType	Maximum-length type	FishBase type code for the maximum-length record.	TL, SL, FL, WD, OT, NG or unspecified; no cross-type

variable	publication label	definition	transformation or note
			conversion was applied.
LengthReferenceCode	Length reference code	FishBase reference code associated with the maximum-length record.	Provenance metadata.
LatitudeGroup	Range-midpoint latitude group	Diagnostic category based on absolute signed range midpoint.	Low, intermediate and high; high n = 5 is descriptive.
SignedNorthernLimit_deg	Signed northern range endpoint	Northern species range endpoint with hemisphere sign (degrees).	Reconstructed from FishBase release 25.04 stocks data.
SignedSouthernLimit_deg	Signed southern range endpoint	Southern species range endpoint with hemisphere sign (degrees).	Reconstructed from FishBase release 25.04 stocks data.
AbsoluteRangeMidpointLatitude_deg	Absolute signed range midpoint	Absolute value of (signed northern endpoint + signed southern endpoint) / 2 (degrees).	Final continuous latitude metric; displayed per 10°.
LatitudinalRangeWidth_deg	Latitudinal range width	Signed northern endpoint minus signed southern endpoint (degrees).	Used in latitude-metric sensitivity analyses.
MaximumAbsoluteRangeLimit_deg	Maximum absolute endpoint	Maximum absolute magnitude of the two signed range endpoints (degrees).	Latitude-metric sensitivity only.
CrossEquatorial	Cross-equatorial indicator	TRUE when the signed range endpoints span the equator.	Used for latitude-metric sensitivity analyses.
LatitudeRangeGroup	Range-midpoint latitude group copy	Explicit copy of the final diagnostic latitude group.	Retained to make the final grouping provenance unambiguous.

All units are included in definitions. The complete canonical data are provided in the reproducibility archive.

Supplementary Table S2. Weight-provenance summary.

Category	n	Definition	Analysis note
Raw Weight available	424	Rows with a non-missing raw FishBase Weight value.	Raw mass contributes to mass-based sensitivity models.
Derived from length-weight parameters	261	Rows without raw Weight for which completed mass was derived as W = aL^b from arithmetic mean FishBase coefficients within SpecCode.	Derived mass is retained only for sensitivity analyses.
Unresolved Weight_g provenance	72	Rows completed by the archived W = 0.01L^3 fallback because their values could not be linked to raw Weight or species-level coefficient records; classified as unresolved in provenance.	These records do not affect primary length-based analyses.

Completed mass is sensitivity evidence; unresolved provenance does not affect length analyses.

Supplementary Table S3. Maximum-length type inventory and TL-only sensitivity.

Length-type inventory

Length type	n	percent	FishBase release	query date
TL	595	78.6	25.0400	2026-07-13
SL	119	15.7	25.0400	2026-07-13
FL	35	4.6	25.0400	2026-07-13
WD	4	0.5	25.0400	2026-07-13
OT	2	0.3	25.0400	2026-07-13
NG	1	0.1	25.0400	2026-07-13
Unspecified	1	0.1	25.0400	2026-07-13

TL-only model

subset	n	term	estimate	95% CI lower	95% CI upper	p
TL-only maximum-length type	595	(Intercept)	1.1481	1.0968	1.1994	<0.001
TL-only maximum-length type	595	x_c	0.5962	0.5261	0.6663	<0.001
TL-only maximum-length type	595	x_c2	-0.3217	-0.4314	-0.2120	<0.001
TL-only maximum-length type	595	depth_1000	0.0727	0.0310	0.1145	<0.001
TL-only maximum-length type	595	latitude_10	0.0193	0.0054	0.0331	0.006

TL = total length; SL = standard length; FL = fork length. No cross-type conversion was applied.

Section S2. FishTree matching and chronogram diagnostics

Supplementary Table S4. FishTree matching, taxonomic coverage and unmatched species.

Matching summary

metric	value	notes
requested scientific names	757	Unique scientific names queried against FishTree.
matched tips	605	Returned tips used in PGLS.
unmatched species	152	The complete list of 152 unmatched species is supplied in the machine-readable workbook and reproducibility archive.

Class-level coverage

taxonomic rank	taxon	canonical n	matched n	unmatched n	matched percent	interpretation
Class	Chondrostei	10	10	0	100.0	fully represented within this canonical taxon
Class	Coelacanthi	1	0	1	0.0	not represented in FishTree-matched PGLS
Class	Elasmobranchii	75	0	75	0.0	not represented in FishTree-matched PGLS
Class	Petromyzonti	3	0	3	0.0	not represented in FishTree-matched PGLS
Class	Teleostei	668	595	73	89.1	partially represented in FishTree-matched PGLS

First 15 unmatched records

SpecCode	FBname	Species	Genus	Family	Order	Class	Species tree	requested tree label	matched using fishtree
37	Lesser sand-eel	Ammodytes marinus	Ammodytes	Ammodytidae	Perciformes/Uranoscopoidei	Teleostei	Ammodytes_marinus	Ammodytes_marinus	FALSE
74	Two-spotted goby	Pomatoschistus flavescens	Pomatoschistus	Gobiidae	Gobiiformes	Teleostei	Pomatoschistus_flavescens	Pomatoschistus_flavescens	FALSE
78	Atlantic sailfish	Istiophorus albicans	Istiophorus	Istiophoridae	Carangiformes	Teleostei	Istiophorus_albicans	Istiophorus_albicans	FALSE
87	Deep-water red snapper	Etelis carbunculus	Etelis	Lutjanidae	Eupercaria/misc	Teleostei	Etelis_carbunculus	Etelis_carbunculus	FALSE
88	Porbeagle	Lamna nasus	Lamna	Lamnidae	Lamniformes	Elasmobranchii	Lamna_nasus	Lamna_nasus	FALSE
138	Greenland shark	Somniosus microcephalus	Somniosus	Somniosidae	Squaliformes	Elasmobranchii	Somniosus_microcephalus	Somniosus_microcephalus	FALSE

SpecCode	FBname	Species	Genus	Family	Order	Class	Species tree	requested tree label	matched using fishtree
139	Piked dogfish	Squalus acanthias	Squalus	Squalidae	Squaliformes	Elasmobranchii	Squalus_acanthias	Squalus_acanthias	FALSE
235	Cisco	Coregonus artedi	Coregonus	Salmonidae	Salmoniformes	Teleostei	Coregonus_artedi	Coregonus_artedi	FALSE
247	Arctic char	Salvelinus alpinus	Salvelinus	Salmonidae	Salmoniformes	Teleostei	Salvelinus_alpinus	Salvelinus_alpinus	FALSE
253	Rainbow smelt	Osmerus mordax	Osmerus	Osmeridae	Osmeriformes	Teleostei	Osmerus_mordax	Osmerus_mordax	FALSE
297	Short-finned eel	Anguilla australis	Anguilla	Anguillidae	Anguilliformes	Teleostei	Anguilla_australis	Anguilla_australis	FALSE
318	Alaska pollock	Gadus chalcogrammus	Gadus	Gadidae	Gadiformes	Teleostei	Gadus_chalcogrammus	Gadus_chalcogrammus	FALSE
445	Red seabream	Pagrus major	Pagrus	Sparidae	Eupercaria/misc	Teleostei	Pagrus_major	Pagrus_major	FALSE
456	Diminutive goby	Lebetus scorpioides	Lebetus	Gobiidae	Gobiiformes	Teleostei	Lebetus_scorpioides	Lebetus_scorpioides	FALSE
464	New Zealand blue cod	Parapercis colias	Parapercis	Pinguipedidae	Perciformes/Uranoscopoidei	Teleostei	Parapercis_colias	Parapercis_colias	FALSE

The complete list of 152 unmatched species and order-level coverage are in the workbook and reproducibility archive. All canonical elasmobranchs were absent from the matched subset.

Supplementary Table S5. FishTree chronogram diagnostics.

Tree version	Tip n	Duplicate tips	Branch lengths	Nonpositive branches	Binary	Polytomies	Root-tip min	Root-tip max	Root-tip range	Relative range	Ultrametric default	Ultrametric tol 1e−7	Max terminal adjustment	Tree source	Decision
FishTree chronogram as returned	605	0.0000	TRUE	0.0000	TRUE	0.0000	333.3698	333.3698	7.000e-06	2.100e-08	FALSE	TRUE	0.0000	FishTree query (archived)	Valid chronogram rerun
Terminal branches extended within numerical tolerance	605	0.0000	TRUE	0.0000	TRUE	0.0000	333.3698	333.3698	1.137e-13	3.410e-16	TRUE	TRUE	7.000e-06	FishTree query (archived)	Valid chronogram rerun

The maximum terminal adjustment was 7 × 10^-6 branch-length units and did not change topology. The tree was queried using fishtree::fishtree_phylogeny(type = 'chronogram'), and the query is archived with the submission package.

Section S3. Functional form and environmental model outputs

Supplementary Table S6. Functional-form comparison, decision and upper-tail support.

Model comparison

Model ID	Dataset	Display model	n	AIC	Adjusted R^2	Nested F p	Quadratic estimate	Quadratic SE	CI lower	CI upper	Quadratic p	Residual RMSE	Trend RMS	CookD > 4/n	BP statistic	BP p	Estimator	AIC role	ΔAIC vs linear
M1 linear	Canonical	Linear length	757	540.831	0.317	NA	NA	NA	NA	NA	NA	0.344	0.0971	37	1.3150	0.251	OLS	Same response; Gaussian comparison	0.000
M2 quadratic	Canonical	Quadratic length	757	497.486	0.356	<0.001	-0.3233	0.0474	-0.4163	-0.2303	<0.001	0.334	0.0378	30	0.0021	0.964	OLS	Same response; Gaussian comparison	-43.345
M3 GAM-ML	Canonical	GAM s(length), k = 4	757	492.601	0.361	NA	NA	NA	NA	NA	NA	0.333	0.0375	NA	NA	NA	ML GAM	AIC sensitivity; same observations	-48.230
M4 GAM-REML	Canonical	GAM s(length), k = 4	757	NA	0.361	NA	NA	NA	NA	NA	NA	0.333	0.0375	NA	NA	NA	REML GAM	Shape sensitivity; AIC not ranked	NA
CS M1 linear	Central support [1.3010, 2.3758]	Linear length	621	426.393	0.138	NA	NA	NA	NA	NA	NA	0.339	0.0759	33	0.6215	0.430	OLS	Same response; Gaussian comparison	0.000
CS M2 quadratic	Central support [1.3010, 2.3758]	Quadratic length	621	398.949	0.176	<0.001	-0.9581	0.1749	-1.3016	-0.6146	<0.001	0.331	0.0205	23	0.0806	0.777	OLS	Same response; Gaussian comparison	-27.445

Display note. Response is log10 maximum longevity and length is centred log10 maximum length. The workbook retains the complete machine-readable model IDs, formulas, estimation roles and comparison fields.

Decision

decision	x center mean log10 length	ΔAIC (quadratic − linear)	nested F p	residual pattern RMS reduction fraction	common support lower log10 length	common support upper log10 length	common support n	full quadratic estimate	full quadratic p	common support quadratic estimate	common support quadratic p	criterion ΔAIC	criterion nested test	criterion residual smooth	criterion common support stability
Quadratic primary functional form	1.7882	-43.345	<0.001	0.6105	1.3010	2.3758	621	-0.3233	1.839e-11	-0.9581	6.268e-08	TRUE	TRUE	TRUE	TRUE

Upper-tail support

quantity	value	interpretation
estimated_zero_derivative_log10_length	2.6872	descriptive location where the fitted quadratic derivative equals zero
estimated_zero_derivative_length_cm	486.6120	descriptive display scale only
species_above_estimated_zero_derivative	15	sample support beyond the fitted attenuation region
species_above_top_1_percent_cutoff	8	descriptive extreme-size support
species_above_top_2_percent_cutoff	16	descriptive extreme-size support

quantity	value	interpretation
CookD_over_4n_above_estimated_zero_derivative	5	influence diagnostic among species beyond the fitted attenuation region
central_common_support_upper_log10_length	2.3758	upper limit of the depth-group central common-support window
estimated_zero_derivative_within_central_common_support	0.0000	1 indicates that the fitted zero-derivative location lies within the central common-support window

RMS of fitted residual trend is distinct from residual RMSE. The ML-GAM enters the AIC sensitivity; REML-GAM is a shape sensitivity and has no cross-framework AIC rank.

Supplementary Table S7. Selected OLS outputs.

Model	Dataset	n	Term	Estimate	SE	95% CI lower	95% CI upper	p	Adjusted R²
OLS final size model	Canonical data, n = 757	757	(Intercept)	1.2293	0.0145	1.2010	1.2577	<0.001	0.356
OLS final size model	Canonical data, n = 757	757	x_c	0.5812	0.0300	0.5223	0.6402	<0.001	0.356
OLS final size model	Canonical data, n = 757	757	x_c2	-0.3233	0.0474	-0.4163	-0.2303	<0.001	0.356
OLS final environmental model	Canonical data, n = 757	757	(Intercept)	1.1429	0.0228	1.0981	1.1876	<0.001	0.379
OLS final environmental model	Canonical data, n = 757	757	x_c	0.5764	0.0304	0.5168	0.6361	<0.001	0.379
OLS final environmental model	Canonical data, n = 757	757	x_c2	-0.3207	0.0471	-0.4132	-0.2282	<0.001	0.379
OLS final environmental model	Canonical data, n = 757	757	depth_1000	0.0692	0.0194	0.0312	0.1072	<0.001	0.379
OLS final environmental model	Canonical data, n = 757	757	latitude_10	0.0200	0.0062	0.0078	0.0323	0.001	0.379
OLS mass environmental sensitivity	Canonical data, n = 757	757	(Intercept)	0.3370	0.0424	0.2537	0.4202	<0.001	0.388
OLS mass environmental sensitivity	Canonical data, n = 757	757	log_Weight	0.2166	0.0104	0.1961	0.2371	<0.001	0.388
OLS mass environmental sensitivity	Canonical data, n = 757	757	depth_1000	0.0665	0.0189	0.0293	0.1036	<0.001	0.388
OLS mass environmental sensitivity	Canonical data, n = 757	757	latitude_10	0.0296	0.0062	0.0175	0.0417	<0.001	0.388

Every OLS row is supplied in the machine-readable workbook.

Supplementary Table S8. Selected PGLS outputs.

Model	Dataset	n	Term	Estimate	SE	95% CI lower	95% CI upper	p	Adjusted R²	Pagel's λ
PGLS final size model	FishTree matched, n = 605	605	(Intercept)	1.2063	0.2863	0.6440	1.7687	<0.001	0.299	0.893
PGLS final size model	FishTree matched, n = 605	605	x_c	0.7134	0.0452	0.6246	0.8022	<0.001	0.299	0.893
PGLS final size model	FishTree matched, n = 605	605	x_c2	-0.1285	0.0700	-0.2659	0.0089	0.067	0.299	0.893
PGLS final environmental model	FishTree matched, n = 605	605	(Intercept)	1.0433	0.2723	0.5085	1.5782	<0.001	0.352	0.887
PGLS final environmental model	FishTree matched, n = 605	605	x_c	0.6693	0.0439	0.5830	0.7555	<0.001	0.352	0.887
PGLS final environmental model	FishTree matched, n = 605	605	x_c2	-0.1397	0.0675	-0.2723	-0.0070	0.039	0.352	0.887
PGLS final environmental model	FishTree matched, n = 605	605	depth_1000	0.1062	0.0225	0.0621	0.1503	<0.001	0.352	0.887
PGLS final environmental model	FishTree matched, n = 605	605	latitude_10	0.0397	0.0081	0.0237	0.0557	<0.001	0.352	0.887
PGLS mass environmental sensitivity	FishTree matched, n = 605	605	(Intercept)	0.3114	0.2691	-0.2170	0.8398	0.248	0.336	0.871
PGLS mass environmental sensitivity	FishTree matched, n = 605	605	log_Weight	0.2131	0.0142	0.1852	0.2411	<0.001	0.336	0.871
PGLS mass environmental sensitivity	FishTree matched, n = 605	605	depth_1000	0.1083	0.0226	0.0639	0.1528	<0.001	0.336	0.871
PGLS mass environmental sensitivity	FishTree matched, n = 605	605	latitude_10	0.0422	0.0082	0.0261	0.0583	<0.001	0.336	0.871

PGLS is restricted to the 605 FishTree-matched species.

Supplementary Table S9. Matched-subset OLS-PGLS comparison.

method	term	estimate	SE	95% CI lower	95% CI upper
Matched OLS	(Intercept)	1.1642	0.0256	1.1138	1.2145
Matched OLS	x_c	0.5968	0.0396	0.5190	0.6746
Matched OLS	x_c2	-0.3944	0.0662	-0.5243	-0.2645
Matched OLS	depth_1000	0.0673	0.0230	0.0221	0.1125
Matched OLS	latitude_10	0.0207	0.0071	0.0068	0.0346
PGLS	(Intercept)	1.0433	0.2723	0.5085	1.5782
PGLS	x_c	0.6693	0.0439	0.5830	0.7555
PGLS	x_c2	-0.1397	0.0675	-0.2723	-0.0070
PGLS	depth_1000	0.1062	0.0225	0.0621	0.1503
PGLS	latitude_10	0.0397	0.0081	0.0237	0.0557

Supplementary Table S10. Quadratic environmental models.

model id	dataset	n	formula	Adjusted R²	AIC	nested F p	term	estimate	SE	95% CI lower	95% CI upper	p
E1_linear	Signed range-midpoint complete cases	757	y ~ x_c + depth_1000 + latitude_10	0.342	514.917	<0.001	(Intercept)	1.0813	0.0215	1.0390	1.1236	<0.001
E1_linear	Signed range-midpoint complete cases	757	y ~ x_c + depth_1000 + latitude_10	0.342	514.917	<0.001	x_c	0.5831	0.0313	0.5216	0.6445	<0.001
E1_linear	Signed range-midpoint complete cases	757	y ~ x_c + depth_1000 + latitude_10	0.342	514.917	<0.001	depth_1000	0.0540	0.0198	0.0152	0.0929	0.006
E1_linear	Signed range-midpoint complete cases	757	y ~ x_c + depth_1000 + latitude_10	0.342	514.917	<0.001	latitude_10	0.0258	0.0064	0.0133	0.0383	<0.001
E2_quadratic	Signed range-midpoint complete cases	757	y ~ x_c + x_c2 + depth_1000 + latitude_10	0.379	471.693	<0.001	(Intercept)	1.1429	0.0228	1.0981	1.1876	<0.001
E2_quadratic	Signed range-midpoint complete cases	757	y ~ x_c + x_c2 + depth_1000 + latitude_10	0.379	471.693	<0.001	x_c	0.5764	0.0304	0.5168	0.6361	<0.001
E2_quadratic	Signed range-midpoint complete cases	757	y ~ x_c + x_c2 + depth_1000 + latitude_10	0.379	471.693	<0.001	x_c2	-0.3207	0.0471	-0.4132	-0.2282	<0.001
E2_quadratic	Signed range-midpoint complete cases	757	y ~ x_c + x_c2 + depth_1000 + latitude_10	0.379	471.693	<0.001	depth_1000	0.0692	0.0194	0.0312	0.1072	<0.001
E2_quadratic	Signed range-midpoint complete cases	757	y ~ x_c + x_c2 + depth_1000 + latitude_10	0.379	471.693	<0.001	latitude_10	0.0200	0.0062	0.0078	0.0323	0.001
CS_E1_linear	Central common support and signed latitude complete cases	621	y ~ x_c + depth_1000 + latitude_10	0.174	401.487	<0.001	(Intercept)	1.1271	0.0237	1.0806	1.1736	<0.001
CS_E1_linear	Central common support and signed latitude complete cases	621	y ~ x_c + depth_1000 + latitude_10	0.174	401.487	<0.001	x_c	0.4916	0.0528	0.3880	0.5953	<0.001
CS_E1_linear	Central common support and signed latitude complete cases	621	y ~ x_c + depth_1000 + latitude_10	0.174	401.487	<0.001	depth_1000	0.0986	0.0226	0.0543	0.1429	<0.001
CS_E1_linear	Central common support and signed latitude complete cases	621	y ~ x_c + depth_1000 + latitude_10	0.174	401.487	<0.001	latitude_10	0.0129	0.0071	-0.0009	0.0268	0.067
CS_E2_quadratic	Central common support and signed latitude complete cases	621	y ~ x_c + x_c2 + depth_1000 + latitude_10	0.211	374.181	<0.001	(Intercept)	1.1954	0.0263	1.1437	1.2470	<0.001
CS_E2_quadratic	Central common support and signed latitude complete cases	621	y ~ x_c + x_c2 + depth_1000 + latitude_10	0.211	374.181	<0.001	x_c	0.5469	0.0526	0.4436	0.6502	<0.001
CS_E2_quadratic	Central common support and signed latitude complete cases	621	y ~ x_c + x_c2 + depth_1000 + latitude_10	0.211	374.181	<0.001	x_c2	-0.9365	0.1716	-1.2736	-0.5994	<0.001
CS_E2_quadratic	Central common support and signed latitude complete cases	621	y ~ x_c + x_c2 + depth_1000 + latitude_10	0.211	374.181	<0.001	depth_1000	0.1007	0.0221	0.0574	0.1440	<0.001
CS_E2_quadratic	Central common support and signed latitude complete cases	621	y ~ x_c + x_c2 + depth_1000 + latitude_10	0.211	374.181	<0.001	latitude_10	0.0101	0.0069	-0.0034	0.0237	0.143

The complete output is supplied in the workbook.

Section S4. Group-slope and quantile diagnostics

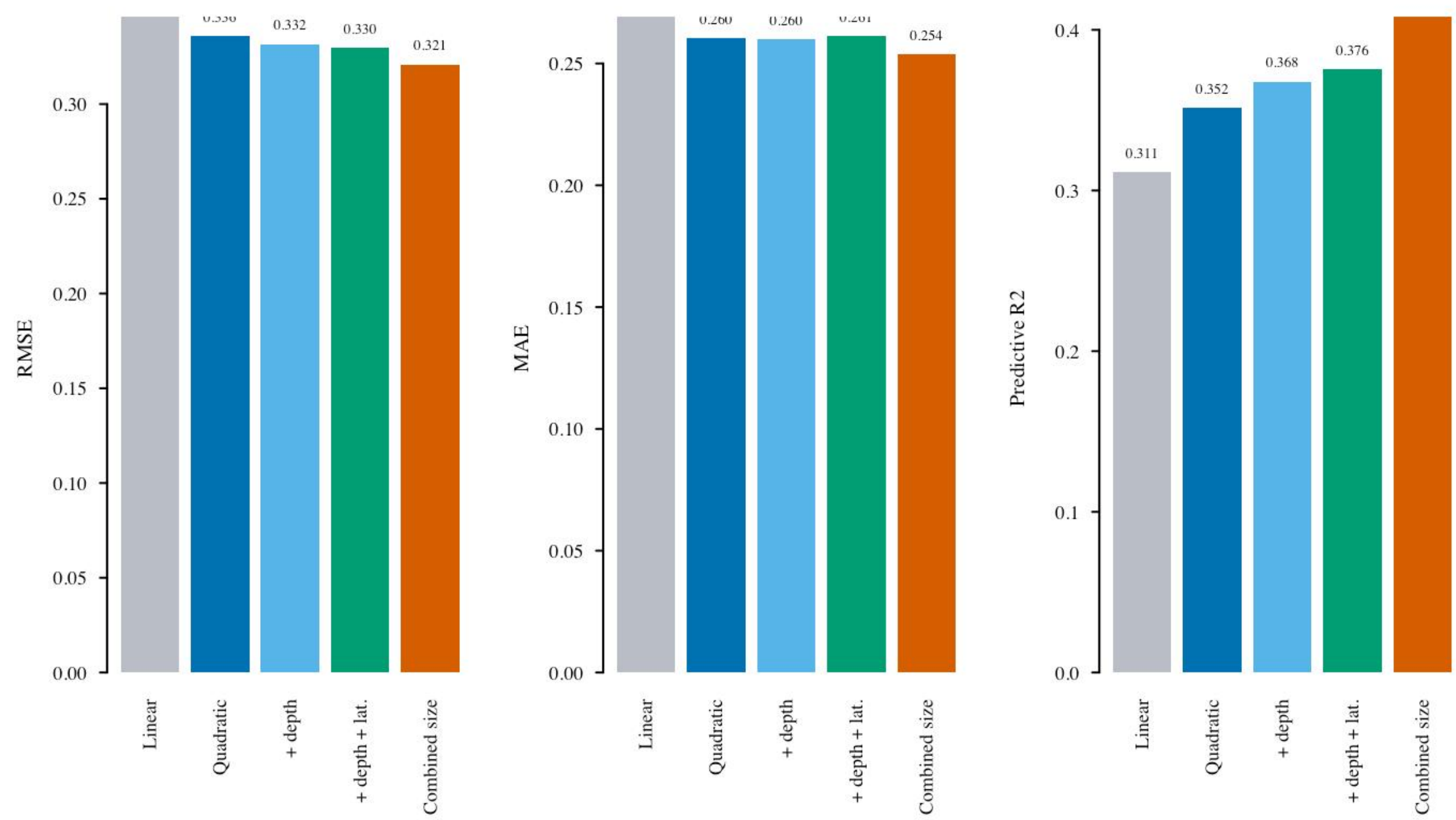

Supplementary Figure S2. Fixed-seed, SpecCode-randomized five-fold diagnostic metrics for the linear comparator, retained quadratic model, environmental additions and combined-size sensitivity. These are internal model checks; complete fold maps and fold-level metrics are archived.

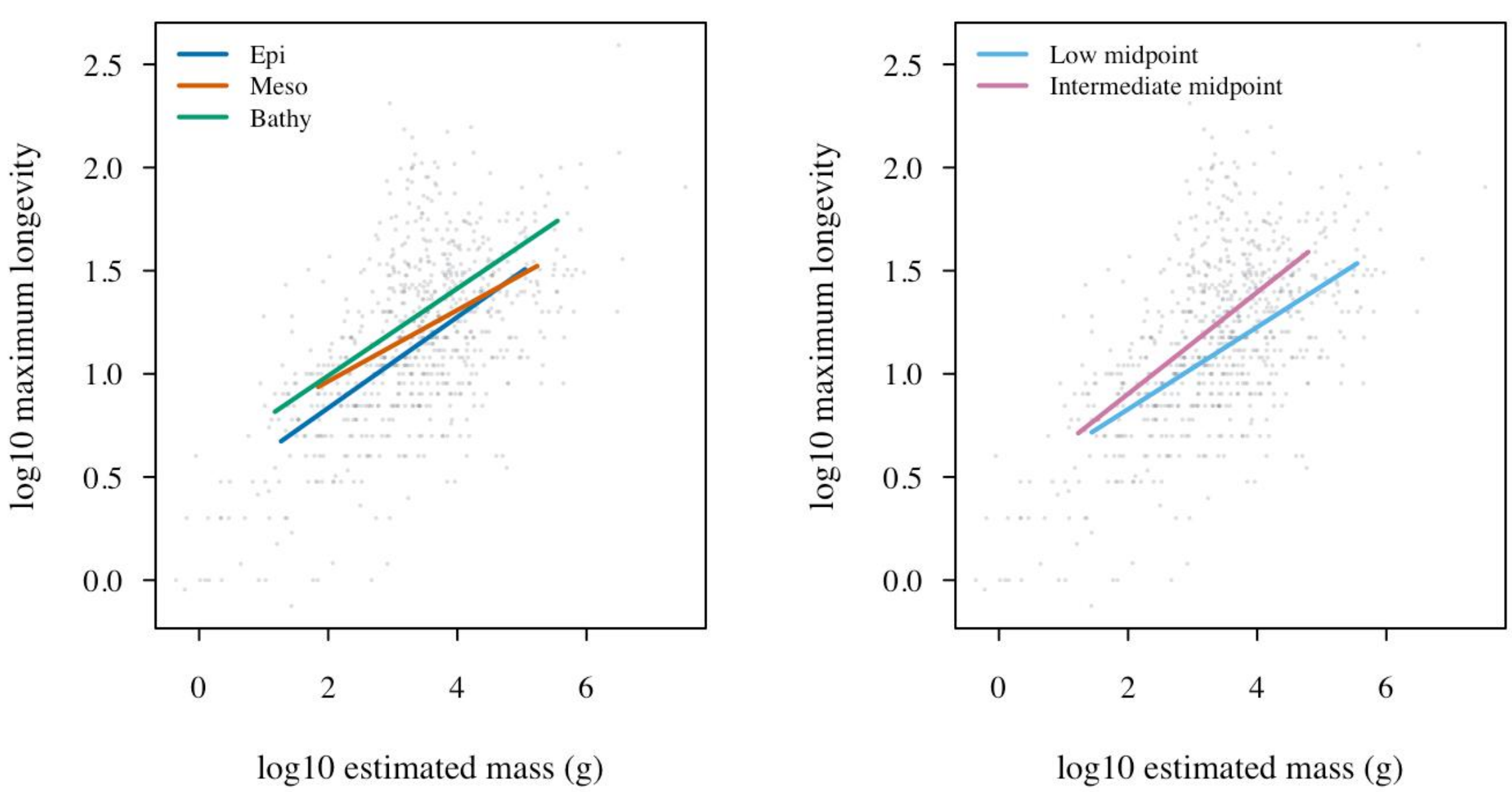

Supplementary Figure S3. Mass-based group sensitivities. Mass relationships are shown separately from primary length models because completed mass was partly derived.

Supplementary Table S11. Common-curvature OLS group-slope contrasts.

row type	family	term	estimate	SE	95% CI lower	95% CI upper	p	FDR q value	n
slope_contrast	Depth group with common curvature	Epipelagic-associated (<200 m) - Mesopelagic-associated (200–<1000 m)	0.0838	0.0715	-0.0566	0.2241	0.242	0.362	757
slope_contrast	Depth group with common curvature	Epipelagic-associated (<200 m) - Bathypelagic-associated (≥1000 m)	-0.0545	0.0803	-0.2121	0.1032	0.498	0.498	757
slope_contrast	Depth group with common curvature	Mesopelagic-associated (200–<1000 m) - Bathypelagic-associated (≥1000 m)	-0.1382	0.0868	-0.3086	0.0322	0.112	0.335	757
slope_contrast	Range-midpoint latitude group with common curvature	Low range-midpoint latitude - Intermediate range-midpoint latitude	-0.1134	0.0629	-0.2369	0.0101	0.072	0.216	757
slope_contrast	Range-midpoint latitude group with common curvature	Low range-midpoint latitude - High range-midpoint latitude	0.6569	1.3659	-2.0244	3.3383	0.631	0.631	757
slope_contrast	Range-midpoint latitude group with common curvature	Intermediate range-midpoint latitude - High range-midpoint latitude	0.7703	1.3656	-1.9105	3.4512	0.573	0.631	757

The high range-midpoint group was descriptive and its contrasts were not interpreted.

Supplementary Table S12. PGLS common-curvature group contrasts.

Model	Contrast	Estimate	SE	95% CI lower	95% CI upper	p	FDR q value	Dataset	tree status
PGLS depth groups with common curvature	Epipelagic-associated (<200 m) - Mesopelagic-associated (200–<1000 m)	-0.0309	0.0748	-0.1777	0.1160	0.680	0.680	FishTree matched	Validated FishTree chronogram; terminal correction within 7e-6 branch-length units
PGLS depth groups with common curvature	Epipelagic-associated (<200 m) - Bathypelagic-associated (≥1000 m)	0.0439	0.0989	-0.1503	0.2381	0.657	0.680	FishTree matched	Validated FishTree chronogram; terminal correction within 7e-6 branch-length units
PGLS depth groups with common curvature	Mesopelagic-associated (200–<1000 m) - Bathypelagic-associated (≥1000 m)	0.0747	0.0907	-0.1034	0.2529	0.410	0.680	FishTree matched	Validated FishTree chronogram; terminal correction within 7e-6 branch-length units
PGLS range-midpoint latitude groups with common curvature	Low range-midpoint latitude - Intermediate range-midpoint latitude	0.0722	0.0675	-0.0604	0.2048	0.286	0.286	FishTree matched low/intermediate range-midpoint groups; high group descriptive only	Validated FishTree chronogram; terminal correction within 7e-6 branch-length units

Supplementary Table S13. Linear quantile slope estimates.

Dataset	Size metric	Tau	Estimate	SE
Canonical, n = 757	Length	0.1000	0.6407	0.0531
Canonical, n = 757	Length	0.2500	0.5855	0.0413
Canonical, n = 757	Length	0.5000	0.5680	0.0363
Canonical, n = 757	Length	0.7500	0.5680	0.0390
Canonical, n = 757	Length	0.9000	0.4274	0.0506
Canonical, n = 757	Length	0.9500	0.3507	0.0931
Canonical, n = 757	Mass	0.1000	0.2276	0.0168
Canonical, n = 757	Mass	0.2500	0.2160	0.0136
Canonical, n = 757	Mass	0.5000	0.2078	0.0114
Canonical, n = 757	Mass	0.7500	0.2191	0.0131
Canonical, n = 757	Mass	0.9000	0.1762	0.0273
Canonical, n = 757	Mass	0.9500	0.1711	0.0129

Supplementary Table S14. Linear quantile bootstrap contrasts and upper-tail support.

Bootstrap contrasts

Dataset	Size metric	Contrast	Estimate	95% CI lower	95% CI upper	p	B	seed	CI type	p value method	quantile engine	FDR q value	Interpretation
Canonical, n = 757	Length	$\beta_{0.90} - \beta_{0.50}$	-0.1406	-0.2358	0.0330	0.128	5000	20260610	percentile 95% bootstrap interval	two-sided sign/tail probability: 2 * min(Pr(contrast <= 0), Pr(contrast >= 0)), capped at 1	quantreg::rq(method = 'br'), quantreg 6.1	0.256	bootstrap interval overlaps zero
Canonical, n = 757	Length	$\beta_{0.95} - \beta_{0.50}$	-0.2173	-0.3537	0.1577	0.288	5000	20260610	percentile 95% bootstrap interval	two-sided sign/tail probability: 2 * min(Pr(contrast <= 0), Pr(contrast >= 0)), capped at 1	quantreg::rq(method = 'br'), quantreg 6.1	0.288	bootstrap interval overlaps zero
Canonical, n = 757	Length	$\beta_{0.90} - \beta OLS$	-0.1524	-0.2226	0.0075	0.068	5000	20260610	percentile 95% bootstrap interval	two-sided sign/tail probability: 2 * min(Pr(contrast <= 0), Pr(contrast >= 0)), capped at 1	quantreg::rq(method = 'br'), quantreg 6.1	0.256	bootstrap interval overlaps zero
Canonical, n = 757	Length	$\beta_{0.95} - \beta OLS$	-0.2291	-0.3507	0.1345	0.249	5000	20260610	percentile 95% bootstrap interval	two-sided sign/tail probability: 2 * min(Pr(contrast <= 0), Pr(contrast >= 0)), capped at 1	quantreg::rq(method = 'br'), quantreg 6.1	0.288	bootstrap interval overlaps zero
Canonical, n = 757	Mass	$\beta_{0.90} - \beta_{0.50}$	-0.0316	-0.0734	0.0409	0.316	5000	20260610	percentile 95% bootstrap interval	two-sided sign/tail probability: 2 * min(Pr(contrast <= 0), Pr(contrast >= 0)), capped at 1	quantreg::rq(method = 'br'), quantreg 6.1	0.633	bootstrap interval overlaps zero
Canonical, n = 757	Mass	$\beta_{0.95} - \beta_{0.50}$	-0.0367	-0.0911	0.0651	0.771	5000	20260610	percentile 95% bootstrap interval	two-sided sign/tail probability: 2 * min(Pr(contrast <= 0), Pr(contrast >= 0)), capped at 1	quantreg::rq(method = 'br'), quantreg 6.1	0.771	bootstrap interval overlaps zero
Canonical, n = 757	Mass	$\beta_{0.90} - \beta OLS$	-0.0363	-0.0723	0.0308	0.196	5000	20260610	percentile 95% bootstrap interval	two-sided sign/tail probability: 2 * min(Pr(contrast <= 0), Pr(contrast >= 0)), capped at 1	quantreg::rq(method = 'br'), quantreg 6.1	0.633	bootstrap interval overlaps zero
Canonical, n = 757	Mass	$\beta_{0.95} - \beta OLS$	-0.0414	-0.0921	0.0528	0.686	5000	20260610	percentile 95% bootstrap interval	two-sided sign/tail probability: 2 * min(Pr(contrast <= 0), Pr(contrast >= 0)), capped at 1	quantreg::rq(method = 'br'), quantreg 6.1	0.771	bootstrap interval overlaps zero

Observations around fitted upper quantiles

size metric	τ	n	target upper fraction	target upper n	observed above fitted quantile	observed on fitted quantile	observed below fitted quantile	engine	interpretation
Length	0.9000	757	0.1000	76	75	2	680	quantreg::rq(method = 'br'), quantreg 6.1	descriptive support count; upper-quantile contrasts remain the inferential source
Length	0.9500	757	0.0500	38	37	2	718	quantreg::rq(method = 'br'), quantreg 6.1	descriptive support count; upper-quantile contrasts remain the inferential source
Mass	0.9000	757	0.1000	76	75	2	680	quantreg::rq(method = 'br'), quantreg 6.1	descriptive support count; upper-quantile contrasts remain the inferential source
Mass	0.9500	757	0.0500	38	37	2	718	quantreg::rq(method = 'br'), quantreg 6.1	descriptive support count; upper-quantile contrasts remain the inferential source

B = 5000; percentile 95% intervals; two-sided sign-tail p-values; FDR within size-metric families.

Supplementary Table S15. Quadratic quantile coefficients and local slope contrasts.

row type	τ	x quantile	x log10 length	term	estimate	95% CI lower	95% CI upper	comparison τ	B	seed	CI method
coefficient	0.5000	NA	NA	(Intercept)	1.2091	1.1736	1.2490	NA	1000	20260713	species-bootstrap percentile 95%
coefficient	0.5000	NA	NA	x_c	0.5905	0.5257	0.6460	NA	1000	20260713	species-bootstrap percentile 95%
coefficient	0.5000	NA	NA	x_c2	-0.3054	-0.4266	-0.2085	NA	1000	20260713	species-bootstrap percentile 95%
coefficient	0.9000	NA	NA	(Intercept)	1.6549	1.6033	1.7107	NA	1000	20260713	species-bootstrap percentile 95%
coefficient	0.9000	NA	NA	x_c	0.5636	0.4450	0.7032	NA	1000	20260713	species-bootstrap percentile 95%
coefficient	0.9000	NA	NA	x_c2	-0.3178	-0.6071	-0.1969	NA	1000	20260713	species-bootstrap percentile 95%
coefficient	0.9500	NA	NA	(Intercept)	1.8206	1.7391	1.9541	NA	1000	20260713	species-bootstrap percentile 95%
coefficient	0.9500	NA	NA	x_c	0.5747	0.3556	0.7360	NA	1000	20260713	species-bootstrap percentile 95%
coefficient	0.9500	NA	NA	x_c2	-0.3578	-0.6524	-0.1652	NA	1000	20260713	species-bootstrap percentile 95%
local_slope_contrast	0.9000	0.2500	1.5563	local slope difference versus τ = 0.50	-0.0212	-0.1521	0.1667	0.5000	1000	20260713	species-bootstrap percentile 95%
local_slope_contrast	0.9500	0.2500	1.5563	local slope difference versus τ = 0.50	0.0084	-0.2717	0.2479	0.5000	1000	20260713	species-bootstrap percentile 95%
local_slope_contrast	0.9000	0.5000	1.7782	local slope difference versus τ = 0.50	-0.0267	-0.1476	0.1201	0.5000	1000	20260713	species-bootstrap percentile 95%
local_slope_contrast	0.9500	0.5000	1.7782	local slope difference versus τ = 0.50	-0.0148	-0.2416	0.1381	0.5000	1000	20260713	species-bootstrap percentile 95%
local_slope_contrast	0.9000	0.7500	2.0170	local slope difference versus τ = 0.50	-0.0326	-0.2005	0.0993	0.5000	1000	20260713	species-bootstrap percentile 95%
local_slope_contrast	0.9500	0.7500	2.0170	local slope difference versus τ = 0.50	-0.0398	-0.2845	0.0669	0.5000	1000	20260713	species-bootstrap percentile 95%

B = 1000; seed = 20260713. Full output is machine-readable.

Supplementary Table S16. Quadratic quantile curve predictions.

First 18 rows

τ	x log10 length	predicted log10 longevity	95% CI lower	95% CI upper	B	seed	CI method
0.5000	0.5911	0.0644	-0.1151	0.2291	1000	20260713	species-bootstrap percentile 95%
0.5000	0.6058	0.0839	-0.0910	0.2445	1000	20260713	species-bootstrap percentile 95%
0.5000	0.6206	0.1032	-0.0672	0.2598	1000	20260713	species-bootstrap percentile 95%
0.5000	0.6353	0.1223	-0.0435	0.2750	1000	20260713	species-bootstrap percentile 95%
0.5000	0.6500	0.1413	-0.0201	0.2902	1000	20260713	species-bootstrap percentile 95%
0.5000	0.6648	0.1602	0.0032	0.3053	1000	20260713	species-bootstrap percentile 95%
0.5000	0.6795	0.1790	0.0259	0.3208	1000	20260713	species-bootstrap percentile 95%
0.5000	0.6943	0.1976	0.0484	0.3364	1000	20260713	species-bootstrap percentile 95%
0.5000	0.7090	0.2161	0.0708	0.3519	1000	20260713	species-bootstrap percentile 95%
0.5000	0.7238	0.2345	0.0929	0.3673	1000	20260713	species-bootstrap percentile 95%
0.5000	0.7385	0.2527	0.1149	0.3826	1000	20260713	species-bootstrap percentile 95%
0.5000	0.7533	0.2708	0.1373	0.3978	1000	20260713	species-bootstrap percentile 95%
0.5000	0.7680	0.2887	0.1586	0.4129	1000	20260713	species-bootstrap percentile 95%
0.5000	0.7828	0.3066	0.1798	0.4279	1000	20260713	species-bootstrap percentile 95%
0.5000	0.7975	0.3243	0.2010	0.4421	1000	20260713	species-bootstrap percentile 95%
0.5000	0.8122	0.3418	0.2222	0.4569	1000	20260713	species-bootstrap percentile 95%
0.5000	0.8270	0.3593	0.2431	0.4716	1000	20260713	species-bootstrap percentile 95%
0.5000	0.8417	0.3766	0.2639	0.4863	1000	20260713	species-bootstrap percentile 95%

All curve rows and uncertainty bands are supplied in the workbook.

Section S5. Common support and sensitivity analyses

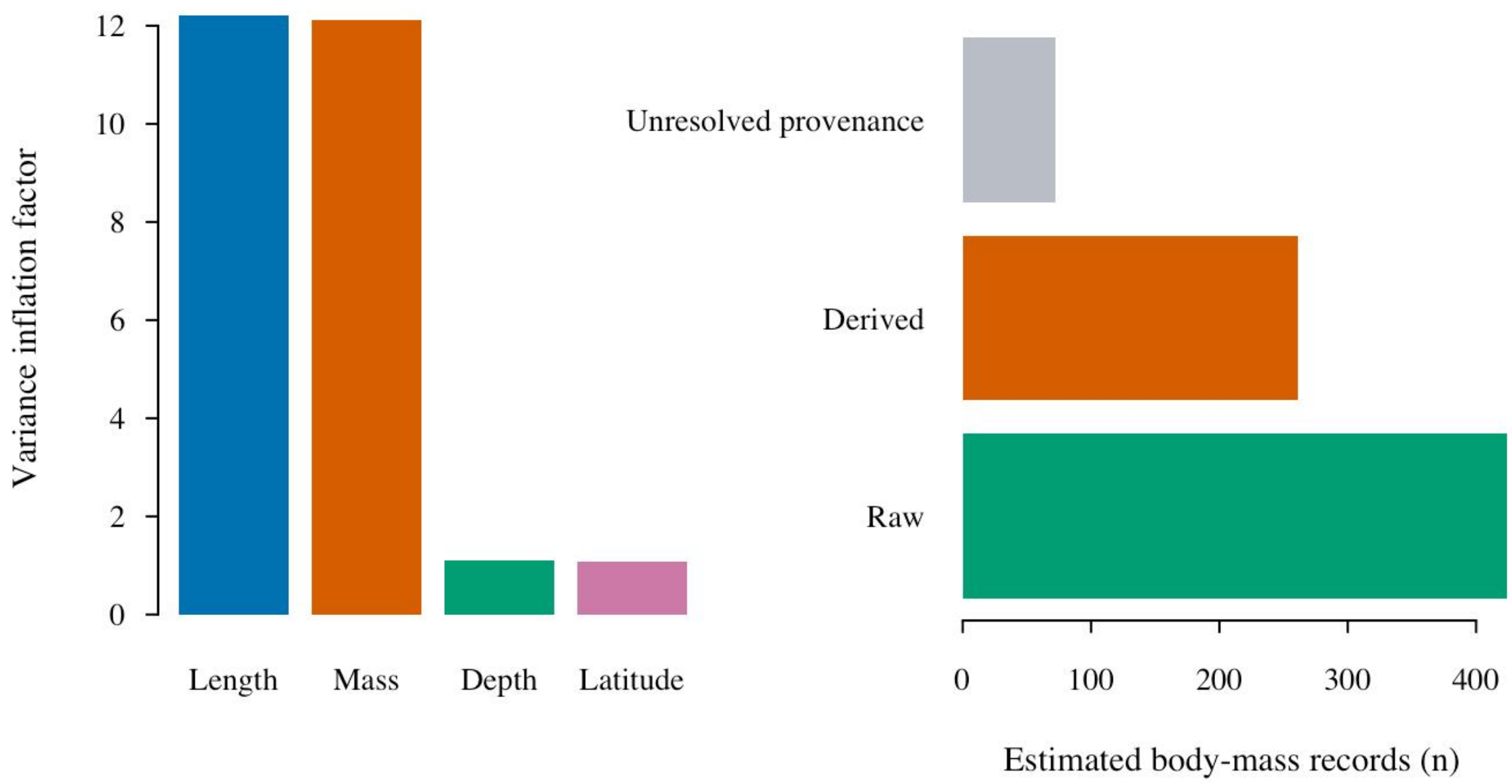

Supplementary Figure S4. Collinearity and body-mass provenance. Length and mass have high variance inflation factors; depth and latitude do not. Provenance labels match Table 1.

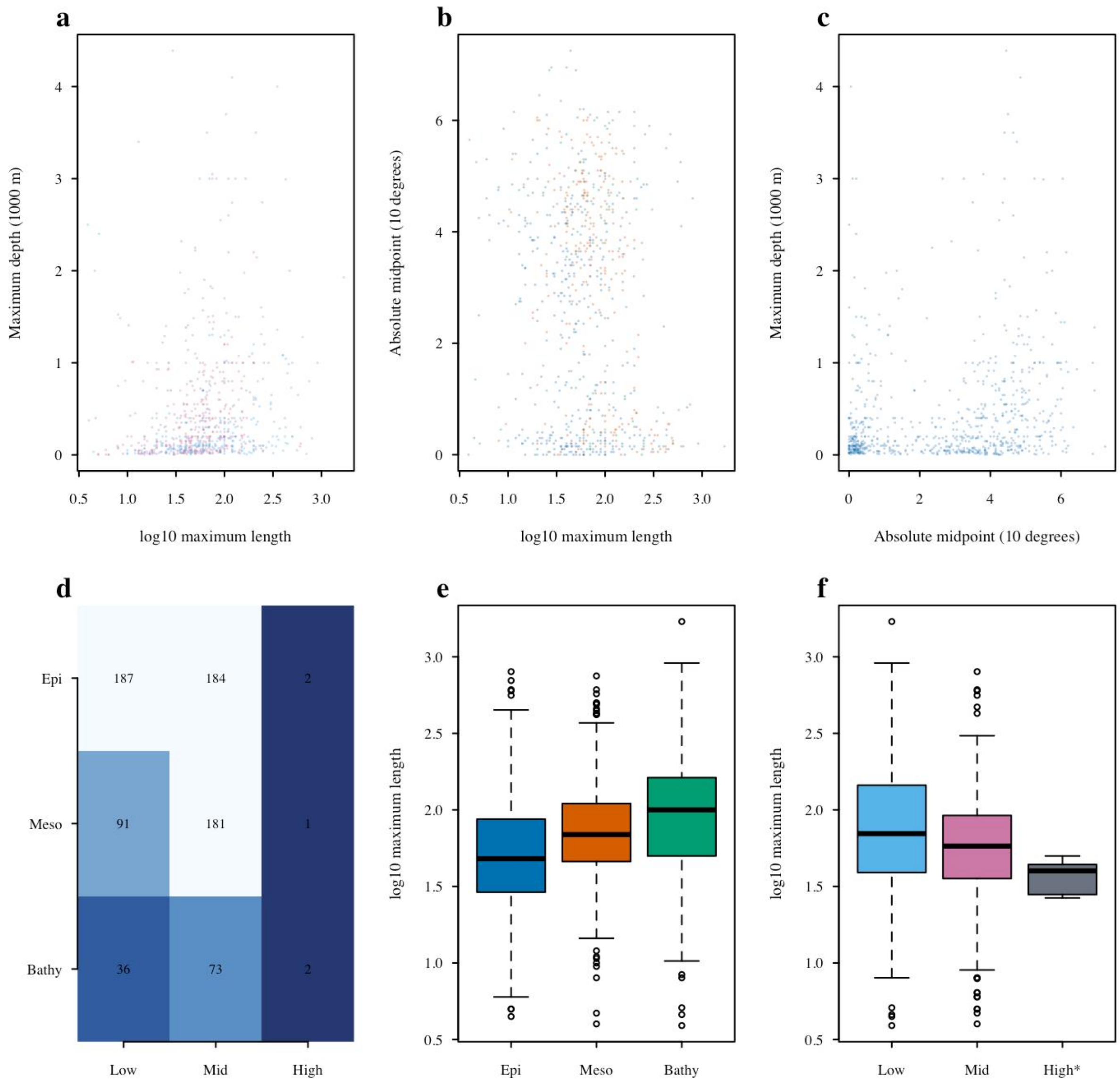

Supplementary Figure S5. Pairwise predictor-overlap and common-support diagnostics. Pairwise scatterplots, group-wise length distributions and cell counts show incomplete depth-by-latitude coverage; the high midpoint group is descriptive.

Supplementary Table S17. Collinearity diagnostics.

Predictor	R^2 against other predictors	VIF
log10 length	0.9181	12.2103
log10 completed mass	0.9175	12.1160
Maximum depth	0.0910	1.1001
Absolute signed range-midpoint latitude	0.0757	1.0819

Supplementary Table S18. Predictor overlap and length-bin occupancy.

Overlap summary

dimension / grouping	category or cell	n	log10 length median	log10 length IQR	maximum depth median / range (m)	absolute range-midpoint latitude median / range (deg)	interpretation status
Depth group	Epipelagic-associated (<200 m)	373	1.6812	1.4624 to 1.9395	64 / 2 to 190	23.0 / 0.0 to 72.5	diagnostic stratum; continuous maximum depth remains primary
Depth group	Mesopelagic-associated (200–<1000 m)	273	1.8388	1.6628 to 2.0414	400 / 200 to 985	36.5 / 0.0 to 69.5	diagnostic stratum; continuous maximum depth remains primary
Depth group	Bathypelagic-associated (≥1000 m)	111	2	1.6990 to 2.2108	1400 / 1000 to 4390	41.5 / 0.0 to 69.5	diagnostic stratum; continuous maximum depth remains primary
Latitude group	Low range-midpoint latitude	314	1.8451	1.5924 to 2.1576	136 / 5 to 4000	3.5 / 0.0 to 23.0	diagnostic stratum; continuous latitude remains primary
Latitude group	Intermediate range-midpoint latitude	438	1.7634	1.5527 to 1.9632	272 / 2 to 4390	42.2 / 23.5 to 64.5	diagnostic stratum; continuous latitude remains primary
Latitude group	High range-midpoint latitude	5	1.6021	1.4472 to 1.6435	700 / 11 to 1383	69.5 / 69.0 to 72.5	sparse/descriptive only
Depth x latitude cell	Epipelagic-associated (<200 m) x Low range-midpoint latitude	187	1.7404	1.5378 to 2.0000	70 / 5 to 190	3.0 / 0.0 to 23.0	diagnostic cell
Depth x latitude cell	Epipelagic-associated (<200 m) x Intermediate range-midpoint latitude	184	1.6370	1.3889 to 1.8451	58 / 2 to 190	38.5 / 23.5 to 62.0	diagnostic cell
Depth x latitude cell	Epipelagic-associated (<200 m) x High range-midpoint latitude	2	1.6712	1.6573 to 1.6851	50 / 11 to 90	70.8 / 69.0 to 72.5	sparse/descriptive only
Depth x latitude cell	Mesopelagic-associated (200–<1000 m) x Low range-midpoint latitude	91	2	1.7782 to 2.3253	350 / 200 to 985	4.5 / 0.0 to 22.0	diagnostic cell
Depth x latitude cell	Mesopelagic-associated (200–<1000 m) x Intermediate range-midpoint latitude	181	1.7993	1.6128 to 1.9614	425 / 200 to 978	44.0 / 23.5 to 64.5	diagnostic cell
Depth x latitude cell	Mesopelagic-associated (200–<1000 m) x High range-midpoint latitude	1	1.4472	1.4472 to 1.4472	700 / 700 to 700	69.5 / 69.5 to 69.5	sparse/descriptive only
Depth x latitude cell	Bathypelagic-associated (≥1000 m) x Low range-midpoint latitude	36	2	1.6680 to 2.4409	1404 / 1000 to 4000	4.0 / 0.0 to 22.0	diagnostic cell
Depth x latitude cell	Bathypelagic-associated (≥1000 m) x Intermediate range-midpoint latitude	73	2	1.7443 to 2.1239	1407 / 1000 to 4390	47.0 / 23.5 to 61.5	diagnostic cell
Depth x latitude cell	Bathypelagic-associated (≥1000 m) x High range-midpoint latitude	2	1.5135	1.4692 to 1.5578	1192 / 1000 to 1383	69.2 / 69.0 to 69.5	sparse/descriptive only

Length-bin occupancy

length bin	log10 length range	Epi n	Meso n	Bathy n	interpretation
Q1	0.591 to 1.477	104	35	16	represented across depth groups
Q2	1.477 to 1.699	93	46	14	represented across depth groups
Q3	1.699 to 1.857	66	69	13	represented across depth groups
Q4	1.857 to 2.086	55	62	32	represented across depth groups
Q5	2.086 to 3.230	55	61	36	represented across depth groups

Supplementary Table S19. Continuous-interaction sensitivity.

model id	model description	n	predictor	estimate	SE	95% CI lower	95% CI upper	p	Adjusted R²	scaling note	interpretation	FDR q value
CI-1	raw maximum depth scaled per 1000 m	757	centered log10 length x maximum depth (per 1000 m)	0.0664	0.0407	-0.0134	0.1461	0.103	0.382	common quadratic length form; maximum depth per 1000 m; absolute signed range-midpoint latitude per 10°	no strong evidence for smooth slope variation	0.103
CI-1	raw maximum depth scaled per 1000 m	757	centered log10 length × absolute signed range-midpoint latitude (per 10°)	0.0265	0.0148	-0.0025	0.0554	0.074	0.382	common quadratic length form; maximum depth per 1000 m; absolute signed range-midpoint latitude per 10°	no strong evidence for smooth slope variation	0.099
CI-2	log10(maximum depth + 1) scale	757	centered log10 length x log10(maximum depth + 1)	0.0928	0.0432	0.0082	0.1774	0.032	0.382	common quadratic length form; log10(maximum depth + 1 m); absolute signed range-midpoint latitude per 10°	unadjusted interaction below 0.05 but not below the FDR threshold	0.095
CI-2	log10(maximum depth + 1) scale	757	centered log10 length × absolute signed range-midpoint latitude (per 10°)	0.0291	0.0147	0.0004	0.0579	0.048	0.382	common quadratic length form; log10(maximum depth + 1 m); absolute signed range-midpoint latitude per 10°	unadjusted interaction below 0.05 but not below the FDR threshold	0.095

FDR q-values determine interpretation within the defined interaction family.

Supplementary Table S20. Common-support and trimmed sensitivities.

Defined subsets

sensitivity id	subset definition	n	retained percent	model formula	predictor	estimate display	SE display	95% CI lower	95% CI upper	p	Adjusted R²	display scale	interpretation
CS-1	Low/intermediate range-midpoint subset; high group excluded as sparse	752	99.3	log_Longevity ~ x_c + x_c2 + depth_1000 + latitude_10	centered log10 length squared	-0.3215	0.0472	-0.4141	-0.2290	<0.001	0.380	original scale	supplementary sensitivity estimate
CS-1	Low/intermediate range-midpoint subset; high group excluded as sparse	752	99.3	log_Longevity ~ x_c + x_c2 + depth_1000 + latitude_10	maximum depth (per 1000 m)	0.0701	0.0194	0.0321	0.1082	<0.001	0.380	per 1000 m	positive depth association retained in sensitivity subset
CS-1	Low/intermediate range-midpoint subset; high group excluded as sparse	752	99.3	log_Longevity ~ x_c + x_c2 + depth_1000 + latitude_10	absolute signed range-midpoint latitude (per 10°)	0.0202	0.0063	0.0078	0.0327	0.001	0.380	per 10°	supplementary sensitivity estimate
CS-2	Intermediate range-midpoint latitude subset	438	57.9	log_Longevity ~ x_c + x_c2 + depth_1000	centered log10 length squared	-0.2486	0.0714	-0.3886	-0.1086	<0.001	0.421	original scale	supplementary sensitivity estimate
CS-2	Intermediate range-midpoint latitude subset	438	57.9	log_Longevity ~ x_c + x_c2 + depth_1000	maximum depth (per 1000 m)	0.0696	0.0220	0.0265	0.1127	0.002	0.421	per 1000 m	positive depth association retained in sensitivity subset
CS-3	Observed common log10 length range across depth groups: 0.650 to 2.875	752	99.3	log_Longevity ~ x_c + x_c2 + depth_1000 + latitude_10	centered log10 length squared	-0.3540	0.0522	-0.4563	-0.2518	<0.001	0.367	original scale	supplementary sensitivity estimate
CS-3	Observed common log10 length range across depth groups: 0.650 to 2.875	752	99.3	log_Longevity ~ x_c + x_c2 + depth_1000 + latitude_10	maximum depth (per 1000 m)	0.0720	0.0196	0.0336	0.1104	<0.001	0.367	per 1000 m	positive depth association retained in sensitivity subset
CS-3	Observed common log10 length range across depth groups: 0.650 to 2.875	752	99.3	log_Longevity ~ x_c + x_c2 + depth_1000 + latitude_10	absolute signed range-midpoint latitude (per 10°)	0.0186	0.0063	0.0063	0.0310	0.003	0.367	per 10°	supplementary sensitivity estimate

Central 5-95% subset

sensitivity id	subset definition	n	retained percent	lower log10 length bound	upper log10 length bound	model formula	predictor	estimate display	SE display	95% CI lower	95% CI upper	p	Adjusted R²	display scale	interpretation
CS-4	Central 5-95% common-support window across depth groups: 1.301 to 2.376 log10 length	621	82.0	1.3010	2.3758	log_Longevity ~ x_c + x_c2 + depth_1000 + latitude_10	centered log10 length squared	-0.9365	0.1716	-1.2729	-0.6001	<0.001	0.211	centered log10 scale	supplementary central common-support estimate
CS-4	Central 5-95% common-support window across depth groups: 1.301 to 2.376 log10 length	621	82.0	1.3010	2.3758	log_Longevity ~ x_c + x_c2 + depth_1000 + latitude_10	maximum depth (per 1000 m)	0.1007	0.0221	0.0575	0.1439	<0.001	0.211	per 1000 m	same qualitative positive depth direction in stricter common-support subset
CS-4	Central 5-95% common-support window across depth groups: 1.301 to 2.376 log10 length	621	82.0	1.3010	2.3758	log_Longevity ~ x_c + x_c2 + depth_1000 + latitude_10	absolute signed range-midpoint latitude (per 10°)	0.0101	0.0069	-0.0034	0.0237	0.143	0.211	per 10°	supplementary central common-support estimate

Supplementary Table S21. Latitude reconstruction and evidence hierarchy.

Reconstruction

metric	value	source	query date
canonical_species	757	FishBase stocks release 25.04; signed range endpoints reconstructed from NorthSouthN/NorthSouthS	2026-07-13
signed_metric_available	757	FishBase stocks release 25.04; signed range endpoints reconstructed from NorthSouthN/NorthSouthS	2026-07-13
signed_metric_missing	0.0000	FishBase stocks release 25.04; signed range endpoints reconstructed from NorthSouthN/NorthSouthS	2026-07-13
cross_equatorial_species	301	FishBase stocks release 25.04; signed range endpoints reconstructed from NorthSouthN/NorthSouthS	2026-07-13
low_group	314	FishBase stocks release 25.04; signed range endpoints reconstructed from NorthSouthN/NorthSouthS	2026-07-13
intermediate_group	438	FishBase stocks release 25.04; signed range endpoints reconstructed from NorthSouthN/NorthSouthS	2026-07-13
high_group	5	FishBase stocks release 25.04; signed range endpoints reconstructed from NorthSouthN/NorthSouthS	2026-07-13
median_absolute_midpoint_deg	31	FishBase stocks release 25.04; signed range endpoints reconstructed from NorthSouthN/NorthSouthS	2026-07-13
median_range_width_deg	40	FishBase stocks release 25.04; signed range endpoints reconstructed from NorthSouthN/NorthSouthS	2026-07-13
median_absolute_legacy_query_difference_deg	0.0000	FishBase stocks release 25.04; signed range endpoints reconstructed from NorthSouthN/NorthSouthS	2026-07-13

Metric coefficients

metric model	n	formula	Adjusted R²	AIC	term	estimate	SE	95% CI lower	95% CI upper	p	interpretation
old_absolute_endpoint_mean_proxy	757	log_Longevity ~ x_c + x_c2 + depth_1000 + legacy_latitude_10	0.372	480.259	legacy_latitude_10	0.0150	0.0115	-0.0076	0.0376	0.193	latitude sensitivity
absolute_signed_range_midpoint	757	log_Longevity ~ x_c + x_c2 + depth_1000 + midpoint_latitude_10	0.379	471.693	midpoint_latitude_10	0.0200	0.0062	0.0078	0.0323	0.001	final latitude metric
maximum_absolute_range_limit	757	log_Longevity ~ x_c + x_c2 + depth_1000 + max_limit_latitude_10	0.372	480.172	max_limit_latitude_10	0.0117	0.0088	-0.0055	0.0289	0.182	latitude sensitivity
midpoint_excluding_cross_equatorial	456	log_Longevity ~ x_c + x_c2 + depth_1000 + midpoint_latitude_10	0.415	250.429	midpoint_latitude_10	0.0286	0.0141	0.0009	0.0564	0.043	latitude sensitivity
midpoint_plus_range_width	757	log_Longevity ~ x_c + x_c2 + depth_1000 + midpoint_latitude_10 + range_width_10	0.380	471.526	midpoint_latitude_10	0.0111	0.0087	-0.0060	0.0282	0.203	latitude sensitivity
midpoint_plus_range_width	757	log_Longevity ~ x_c + x_c2 + depth_1000 + midpoint_latitude_10 + range_width_10	0.380	471.526	range_width_10	-0.0094	0.0064	-0.0219	0.0032	0.143	latitude sensitivity

Cross-analysis evidence summary

evidence id	evidence type	analysis context	n	estimate	SE	95% CI lower	95% CI upper	p	predictive R² change	interpretation	evidence level	overall reading
metric_old_absolute_endpoint_mean_proxy	range-based latitude metric sensitivity	old_absolute_endpoint_mean_proxy	757	0.0150	0.0115	-0.0076	0.0376	0.193	NA	supplementary metric sensitivity; range endpoints are not occurrence-weighted exposure	alternative range-proxy sensitivity	Latitude is exploratory and metric-dependent; it is not weighted equally with the more consistent maximum-depth association.
metric_absolute_signed_range_midpoint	range-based latitude metric sensitivity	absolute_signed_range_midpoint	757	0.0200	0.0062	0.0078	0.0323	0.001	NA	supplementary metric sensitivity; range endpoints are not occurrence-weighted exposure	primary range-proxy coefficient	Latitude is exploratory and metric-dependent; it is not weighted equally with the more consistent maximum-depth association.
metric_maximum_absolute_range_limit	range-based latitude metric sensitivity	maximum_absolute_range_limit	757	0.0117	0.0088	-0.0055	0.0289	0.182	NA	supplementary metric sensitivity; range endpoints are not occurrence-weighted exposure	alternative range-proxy sensitivity	Latitude is exploratory and metric-dependent; it is not weighted equally with the more consistent maximum-depth association.
metric_midpoint_excluding_cross_equatorial	range-based latitude metric sensitivity	midpoint_excluding_cross_equatorial	456	0.0286	0.0141	0.0009	0.0564	0.043	NA	supplementary metric sensitivity; range endpoints are not occurrence-weighted exposure	alternative range-proxy sensitivity	Latitude is exploratory and metric-dependent; it is not weighted equally with the more consistent maximum-depth association.
metric_midpoint_plus_range_width	range-based latitude metric sensitivity	midpoint_plus_range_width	757	0.0111	0.0087	-0.0060	0.0282	0.203	NA	supplementary metric sensitivity; range endpoints are not occurrence-weighted exposure	alternative range-proxy sensitivity	Latitude is exploratory and metric-dependent; it is not weighted equally with the more consistent maximum-depth association.
common_support_CS-1	common-support sensitivity	Low/intermediate range-midpoint subset; high group excluded as sparse	752	0.0202	0.0063	0.0078	0.0327	0.001	NA	supplementary subset estimate	supplementary sensitivity or diagnostic	Latitude is exploratory and metric-dependent; it is not weighted equally with the

evidence id	evidence type	analysis context	n	estimate	SE	95% CI lower	95% CI upper	p	predictive R² change	interpretation	evidence level	overall reading
												more consistent maximum-depth association.
common_support_CS-3	common-support sensitivity	Observed common log10 length range across depth groups: 0.650 to 2.875	752	0.0186	0.0063	0.0063	0.0310	0.003	NA	supplementary subset estimate	supplementary sensitivity or diagnostic	Latitude is exploratory and metric-dependent; it is not weighted equally with the more consistent maximum-depth association.
common_support_CS-4	central common-support sensitivity	Central 5-95% common-support window across depth groups: 1.301 to 2.376 log10 length	621	0.0101	0.0069	-0.0034	0.0237	0.143	NA	supplementary central-window estimate	supplementary sensitivity or diagnostic	Latitude is exploratory and metric-dependent; it is not weighted equally with the more consistent maximum-depth association.
pgls_matched_subset	FishTree-matched PGLS sensitivity	605-species matched subset; all canonical elasmobranchs excluded	605	0.0397	0.0081	0.0237	0.0557	<0.001	NA	matched-subset branch-length covariance sensitivity	supplementary sensitivity or diagnostic	Latitude is exploratory and metric-dependent; it is not weighted equally with the more consistent maximum-depth association.
validation_random_species	internal transportability diagnostic	species-random five-fold diagnostic	757	NA	NA	NA	NA	NA	0.008	change in pooled predictive R² after adding the final latitude proxy to size plus depth; not an external validation	supplementary sensitivity or diagnostic	Latitude is exploratory and metric-dependent; it is not weighted equally with the more consistent maximum-depth association.
validation_family_blocked	internal transportability diagnostic	Family-blocked five-fold diagnostic	757	NA	NA	NA	NA	NA	-0.048	change in pooled predictive R² after adding the final latitude proxy to size plus depth; not an external validation	supplementary sensitivity or diagnostic	Latitude is exploratory and metric-dependent; it is not weighted equally with the more consistent maximum-depth association.
validation_order_blocked	internal transportability diagnostic	Order-blocked five-fold diagnostic	757	NA	NA	NA	NA	NA	-0.065	change in pooled predictive R² after adding the final latitude proxy to size plus depth; not	supplementary sensitivity or diagnostic	Latitude is exploratory and metric-dependent; it is not weighted equally with the more consistent

evidence id	evidence type	analysis context	n	estimate	SE	95% CI lower	95% CI upper	p	predictive R^2 change	interpretation	evidence level	overall reading
										an external validation		maximum-depth association.
taxonomy_teleostei_only	Teleostei-only model	Teleostei species only	668	0.0154	0.0067	0.0024	0.0285	0.021	NA	supplementary taxonomic-composition sensitivity; not primary inference	supplementary sensitivity or diagnostic	Latitude is exploratory and metric-dependent; it is not weighted equally with the more consistent maximum-depth association.
taxonomy_broad_class_adjusted	Broad-Class fixed-effects model	all canonical species; Teleostei reference, Elasmobranchii and Other fishes fixed effects	757	0.0170	0.0063	0.0046	0.0295	0.007	NA	supplementary taxonomic-composition sensitivity; not primary inference	supplementary sensitivity or diagnostic	Latitude is exploratory and metric-dependent; it is not weighted equally with the more consistent maximum-depth association.
taxonomy_family_clustered_CR2	Family-clustered robust uncertainty	all canonical species clustered across complete Family labels	757	0.0200	0.0197	-0.0197	0.0598	0.316	NA	supplementary clustered-uncertainty sensitivity; coefficient estimates equal the canonical OLS fit	supplementary sensitivity or diagnostic	Latitude is exploratory and metric-dependent; it is not weighted equally with the more consistent maximum-depth association.

Latitude is a range-based proxy. The evidence summary includes common-support, PGLS, blocked-validation and clustered-uncertainty results.

Section S6. Diagnostic validation and reproducibility

Supplementary Table S22. Fixed-seed species-random diagnostic validation.

Fold, pooled and fold-variability metrics

row type	scheme	model id	fold	n	groups in test	RMSE	MAE	Predictive R^2	seed
fold	species-random	final_form_size_only	1	152	152	0.365	0.281	0.403	20260713
fold	species-random	final_form_size_only	2	152	152	0.336	0.257	0.313	20260713
fold	species-random	final_form_size_only	3	151	151	0.314	0.242	0.306	20260713
fold	species-random	final_form_size_only	4	151	151	0.318	0.250	0.486	20260713
fold	species-random	final_form_size_only	5	151	151	0.344	0.272	0.175	20260713
pooled	species-random	final_form_size_only	NA	757	757	0.336	0.260	0.352	20260713
fold_SD	species-random	final_form_size_only	NA	757	757	0.021	0.016	0.117	20260713
fold	species-random	final_form_size_depth	1	152	152	0.360	0.283	0.419	20260713
fold	species-random	final_form_size_depth	2	152	152	0.332	0.257	0.329	20260713
fold	species-random	final_form_size_depth	3	151	151	0.313	0.241	0.311	20260713
fold	species-random	final_form_size_depth	4	151	151	0.312	0.248	0.505	20260713
fold	species-random	final_form_size_depth	5	151	151	0.339	0.271	0.199	20260713
pooled	species-random	final_form_size_depth	NA	757	757	0.332	0.260	0.368	20260713
fold_SD	species-random	final_form_size_depth	NA	757	757	0.020	0.017	0.116	20260713
fold	species-random	final_form_size_depth_latitude	1	152	152	0.356	0.282	0.431	20260713
fold	species-random	final_form_size_depth_latitude	2	152	152	0.332	0.259	0.326	20260713
fold	species-random	final_form_size_depth_latitude	3	151	151	0.311	0.246	0.317	20260713
fold	species-random	final_form_size_depth_latitude	4	151	151	0.307	0.248	0.519	20260713
fold	species-random	final_form_size_depth_latitude	5	151	151	0.338	0.270	0.206	20260713
pooled	species-random	final_form_size_depth_latitude	NA	757	757	0.330	0.261	0.376	20260713
fold_SD	species-random	final_form_size_depth_latitude	NA	757	757	0.020	0.015	0.119	20260713

Unique SpecCode values were sorted, randomly permuted with seed 20260713 and allocated cyclically to five folds. These metrics are internal diagnostics.

Supplementary Table S23. Taxonomy-blocked diagnostic validation.

Family-blocked metrics

row type	scheme	model id	fold	n	groups in test	RMSE	MAE	Predictive R²	seed
fold	family-blocked	final_form_size_only	1	187	34	0.459	0.379	0.091	20260713
fold	family-blocked	final_form_size_only	2	133	33	0.352	0.278	0.408	20260713
fold	family-blocked	final_form_size_only	3	138	33	0.287	0.217	0.263	20260713
fold	family-blocked	final_form_size_only	4	147	33	0.284	0.218	0.423	20260713
fold	family-blocked	final_form_size_only	5	152	33	0.268	0.208	0.280	20260713
pooled	family-blocked	final_form_size_only	NA	757	166	0.345	0.266	0.316	20260713
fold_SD	family-blocked	final_form_size_only	NA	757	166	0.079	0.072	0.134	20260713
fold	family-blocked	final_form_size_depth	1	187	34	0.452	0.371	0.120	20260713
fold	family-blocked	final_form_size_depth	2	133	33	0.348	0.277	0.424	20260713
fold	family-blocked	final_form_size_depth	3	138	33	0.278	0.214	0.310	20260713
fold	family-blocked	final_form_size_depth	4	147	33	0.289	0.222	0.400	20260713
fold	family-blocked	final_form_size_depth	5	152	33	0.284	0.225	0.191	20260713
pooled	family-blocked	final_form_size_depth	NA	757	166	0.344	0.268	0.321	20260713
fold_SD	family-blocked	final_form_size_depth	NA	757	166	0.074	0.066	0.131	20260713
fold	family-blocked	final_form_size_depth_latitude	1	187	34	0.453	0.372	0.117	20260713
fold	family-blocked	final_form_size_depth_latitude	2	133	33	0.342	0.274	0.444	20260713
fold	family-blocked	final_form_size_depth_latitude	3	138	33	0.280	0.218	0.297	20260713
fold	family-blocked	final_form_size_depth_latitude	4	147	33	0.285	0.219	0.416	20260713
fold	family-blocked	final_form_size_depth_latitude	5	152	33	0.354	0.297	-0.264	20260713
pooled	family-blocked	final_form_size_depth_latitude	NA	757	166	0.356	0.282	0.273	20260713
fold_SD	family-blocked	final_form_size_depth_latitude	NA	757	166	0.070	0.064	0.291	20260713

Order-blocked metrics

row type	scheme	model id	fold	n	groups in test	RMSE	MAE	Predictive R²	seed
fold	order-blocked	final_form_size_only	1	224	13	0.290	0.221	0.290	20260813
fold	order-blocked	final_form_size_only	2	106	12	0.339	0.271	0.198	20260813
fold	order-blocked	final_form_size_only	3	124	12	0.278	0.211	0.553	20260813
fold	order-blocked	final_form_size_only	4	138	12	0.298	0.241	0.306	20260813
fold	order-blocked	final_form_size_only	5	165	12	0.462	0.379	0.272	20260813
pooled	order-blocked	final_form_size_only	NA	757	61	0.341	0.264	0.331	20260813
fold_SD	order-blocked	final_form_size_only	NA	757	61	0.075	0.068	0.135	20260813
fold	order-blocked	final_form_size_depth	1	224	13	0.288	0.224	0.301	20260813
fold	order-blocked	final_form_size_depth	2	106	12	0.332	0.270	0.231	20260813
fold	order-blocked	final_form_size_depth	3	124	12	0.291	0.227	0.510	20260813
fold	order-blocked	final_form_size_depth	4	138	12	0.290	0.227	0.344	20260813
fold	order-blocked	final_form_size_depth	5	165	12	0.456	0.377	0.291	20260813
pooled	order-blocked	final_form_size_depth	NA	757	61	0.338	0.265	0.342	20260813
fold_SD	order-blocked	final_form_size_depth	NA	757	61	0.072	0.065	0.105	20260813
fold	order-blocked	final_form_size_depth_latitude	1	224	13	0.334	0.268	0.058	20260813
fold	order-blocked	final_form_size_depth_latitude	2	106	12	0.322	0.261	0.276	20260813
fold	order-blocked	final_form_size_depth_latitude	3	124	12	0.313	0.255	0.433	20260813
fold	order-blocked	final_form_size_depth_latitude	4	138	12	0.287	0.223	0.357	20260813
fold	order-blocked	final_form_size_depth_latitude	5	165	12	0.464	0.385	0.263	20260813
pooled	order-blocked	final_form_size_depth_latitude	NA	757	61	0.355	0.282	0.277	20260813
fold_SD	order-blocked	final_form_size_depth_latitude	NA	757	61	0.069	0.062	0.140	20260813

Fold composition

scheme	fold	species n	taxonomic groups n	total taxonomic groups	minimum fold n	maximum fold n	fold size range	interpretation
species-random	1	152	152	757	151	152	1	species-random folds balanced within one species
species-random	2	152	152	757	151	152	1	species-random folds balanced within one species
species-random	3	151	151	757	151	152	1	species-random folds balanced within one species
species-random	4	151	151	757	151	152	1	species-random folds balanced within one species
species-random	5	151	151	757	151	152	1	species-random folds balanced within one species
family-blocked	1	187	34	166	133	187	54	whole-family blocking; approximate greedy size balance
family-blocked	2	133	33	166	133	187	54	whole-family blocking; approximate greedy size balance
family-blocked	3	138	33	166	133	187	54	whole-family blocking; approximate greedy size balance
family-blocked	4	147	33	166	133	187	54	whole-family blocking; approximate greedy size balance
family-blocked	5	152	33	166	133	187	54	whole-family blocking; approximate greedy size balance
order-blocked	1	224	13	61	106	224	118	whole-order blocking; fold sizes are necessarily imbalanced
order-blocked	2	106	12	61	106	224	118	whole-order blocking; fold sizes are necessarily imbalanced
order-blocked	3	124	12	61	106	224	118	whole-order blocking; fold sizes are necessarily imbalanced
order-blocked	4	138	12	61	106	224	118	whole-order blocking; fold sizes are necessarily imbalanced
order-blocked	5	165	12	61	106	224	118	whole-order blocking; fold sizes are necessarily imbalanced

Fold-integrity checks

scheme	species n	unique SpecCode n	fold n	minimum fold n	maximum fold n	cross fold group leakage n	missing group n	status
species-random	757	757	5	151	152	0	0	PASS
family-blocked	757	757	5	133	187	0	0	PASS
order-blocked	757	757	5	106	224	0	0	PASS

Whole families or orders remained in one fold. Order-blocked folds were necessarily unbalanced because order sizes differed.

Supplementary Table S24. Grouped PGLS λ sensitivity.

Fixed and independently estimated λ models

grouping	λ mode	n	Pagel's λ	term or contrast	estimate	SE	95% CI lower	95% CI upper	p	FDR q value	interpretation
depth	Fixed primary λ	605	0.887	(Intercept)	1.1728	0.2779	0.6269	1.7186	<0.001	NA	supplementary coefficient
depth	Fixed primary λ	605	0.887	x_c	0.6730	0.0589	0.5574	0.7886	<0.001	NA	supplementary coefficient
depth	Fixed primary λ	605	0.887	x_c2	-0.1486	0.0699	-0.2860	-0.0113	0.034	NA	supplementary coefficient
depth	Fixed primary λ	605	0.887	Meso vs Epi	0.0796	0.0265	0.0275	0.1316	0.003	NA	supplementary coefficient
depth	Fixed primary λ	605	0.887	Bathy vs Epi	0.2019	0.0425	0.1184	0.2855	<0.001	NA	supplementary coefficient
depth	Fixed primary λ	605	0.887	Centred length × Meso	0.0309	0.0748	-0.1160	0.1777	0.680	0.680	supplementary coefficient
depth	Fixed primary λ	605	0.887	Centred length × Bathy	-0.0439	0.0989	-0.2381	0.1503	0.657	0.680	supplementary coefficient
depth	Fixed primary λ	605	0.887	Epipelagic-associated (<200 m) - Mesopelagic-associated (200–<1000 m)	-0.0309	0.0748	-0.1777	0.1160	0.680	0.680	contrast not below the within-model FDR threshold
depth	Fixed primary λ	605	0.887	Epipelagic-associated (<200 m) - Bathypelagic-associated (≥1000 m)	0.0439	0.0989	-0.1503	0.2381	0.657	0.680	contrast not below the within-model FDR threshold
depth	Fixed primary λ	605	0.887	Mesopelagic-associated (200–<1000 m) - Bathypelagic-associated (≥1000 m)	0.0747	0.0907	-0.1034	0.2529	0.410	0.680	contrast not below the within-model FDR threshold
depth	Independently estimated λ	605	0.881	(Intercept)	1.1734	0.2737	0.6359	1.7110	<0.001	NA	supplementary coefficient
depth	Independently estimated λ	605	0.881	x_c	0.6714	0.0588	0.5559	0.7868	<0.001	NA	supplementary coefficient
depth	Independently estimated λ	605	0.881	x_c2	-0.1504	0.0699	-0.2876	-0.0132	0.032	NA	supplementary coefficient
depth	Independently	605	0.881	Meso vs Epi	0.0797	0.0265	0.0276	0.1318	0.003	NA	supplementary

grouping	λ mode	n	Pagel's λ	term or contrast	estimate	SE	95% CI lower	95% CI upper	p	FDR q value	interpretation
	estimated λ										coefficient
depth	Independently estimated λ	605	0.881	Bathy vs Epi	0.2031	0.0425	0.1196	0.2866	<0.001	NA	supplementary coefficient
depth	Independently estimated λ	605	0.881	Centred length × Meso	0.0302	0.0748	-0.1167	0.1771	0.687	0.687	supplementary coefficient
depth	Independently estimated λ	605	0.881	Centred length × Bathy	-0.0434	0.0988	-0.2374	0.1506	0.661	0.687	supplementary coefficient
depth	Independently estimated λ	605	0.881	Epipelagic-associated (<200 m) - Mesopelagic-associated (200–<1000 m)	-0.0302	0.0748	-0.1771	0.1167	0.687	0.687	contrast not below the within-model FDR threshold
depth	Independently estimated λ	605	0.881	Epipelagic-associated (<200 m) - Bathypelagic-associated (≥1000 m)	0.0434	0.0988	-0.1506	0.2374	0.661	0.687	contrast not below the within-model FDR threshold
depth	Independently estimated λ	605	0.881	Mesopelagic-associated (200–<1000 m) - Bathypelagic-associated (≥1000 m)	0.0736	0.0908	-0.1048	0.2519	0.418	0.687	contrast not below the within-model FDR threshold
latitude	Fixed primary λ	602	0.887	(Intercept)	1.1344	0.2799	0.5846	1.6842	<0.001	NA	supplementary coefficient
latitude	Fixed primary λ	602	0.887	x_c	0.7540	0.0607	0.6347	0.8733	<0.001	NA	supplementary coefficient
latitude	Fixed primary λ	602	0.887	x_c2	-0.1223	0.0694	-0.2586	0.0141	0.079	NA	supplementary coefficient
latitude	Fixed primary λ	602	0.887	Intermediate vs low midpoint	0.1097	0.0298	0.0512	0.1682	<0.001	NA	supplementary coefficient
latitude	Fixed primary λ	602	0.887	Centred length × intermediate midpoint	-0.0722	0.0675	-0.2048	0.0604	0.286	0.286	supplementary coefficient
latitude	Fixed primary λ	602	0.887	Low range-midpoint latitude - Intermediate range-midpoint latitude	0.0722	0.0675	-0.0604	0.2048	0.286	0.286	contrast not below the within-model FDR threshold
latitude	Independently estimated λ	602	0.886	(Intercept)	1.1344	0.2795	0.5855	1.6833	<0.001	NA	supplementary coefficient
latitude	Independently estimated λ	602	0.886	x_c	0.7538	0.0607	0.6345	0.8730	<0.001	NA	supplementary coefficient
latitude	Independently estimated λ	602	0.886	x_c2	-0.1224	0.0694	-0.2587	0.0140	0.078	NA	supplementary coefficient
latitude	Independently estimated λ	602	0.886	Intermediate vs low midpoint	0.1097	0.0298	0.0513	0.1682	<0.001	NA	supplementary coefficient
latitude	Independently estimated λ	602	0.886	Centred length × intermediate midpoint	-0.0721	0.0675	-0.2047	0.0606	0.286	0.286	supplementary coefficient
latitude	Independently estimated λ	602	0.886	Low range-midpoint latitude - Intermediate range-midpoint latitude	0.0721	0.0675	-0.0606	0.2047	0.286	0.286	contrast not below the within-model FDR threshold

Grouped contrasts are supplementary; FDR was applied within each model and λ specification.

Supplementary Table S25. Taxonomic-composition and Family-clustered sensitivity.

Coefficient sensitivities

analysis type	subset definition	n	cluster count	term	estimate	SE	df	95% CI lower	95% CI upper	p	uncertainty method
Canonical OLS reference	all 757 canonical species	757	NA	depth_1000	0.0692	0.0194	752	0.0312	0.1072	<0.001	model-based OLS standard errors
Canonical OLS reference	all 757 canonical species	757	NA	latitude_10	0.0200	0.0062	752	0.0078	0.0323	0.001	model-based OLS standard errors
Teleostei-only model	Teleostei species only	668	NA	depth_1000	0.0832	0.0218	663	0.0405	0.1260	<0.001	model-based OLS standard errors
Teleostei-only model	Teleostei species only	668	NA	latitude_10	0.0154	0.0067	663	0.0024	0.0285	0.021	model-based OLS standard errors
Broad-Class fixed-effects model	all canonical species; Teleostei reference, Elasmobranchii and Other fishes fixed effects	757	NA	depth_1000	0.0720	0.0193	750	0.0341	0.1100	<0.001	model-based OLS standard errors
Broad-Class fixed-effects model	all canonical species; Teleostei reference, Elasmobranchii and Other fishes fixed effects	757	NA	latitude_10	0.0170	0.0063	750	0.0046	0.0295	0.007	model-based OLS standard errors
Family-clustered robust uncertainty	all canonical species clustered across complete Family labels	757	166	depth_1000	0.0692	0.0285	18.5474	0.0094	0.1289	0.026	clubSandwich CR2, Family clusters, Satterthwaite degrees of freedom
Family-clustered robust uncertainty	all canonical species clustered across complete Family labels	757	166	latitude_10	0.0200	0.0197	43.3803	-0.0197	0.0598	0.316	clubSandwich CR2, Family clusters, Satterthwaite degrees of freedom
Elasmobranchii-only support sensitivity	75 elasmobranch species; limited within-class support, not a primary subgroup model	75	NA	depth_1000	0.1670	0.0377	70	0.0917	0.2423	<0.001	model-based OLS standard errors
Elasmobranchii-only support sensitivity	75 elasmobranch species; limited within-class support, not a primary subgroup model	75	NA	latitude_10	0.0237	0.0179	70	-0.0121	0.0594	0.191	model-based OLS standard errors

Predictor support

subset	n	family clusters	log10 length min	log10 length max	maximum depth min m	maximum depth max m	absolute midpoint latitude min deg	absolute midpoint latitude max deg	interpretation
Canonical	757	166	0.5911	3.2304	2	4390	0.0000	72.5000	primary canonical support
Teleostei	668	139	0.5911	2.6990	2	4390	0.0000	72.5000	large within-class sensitivity subset
Elasmobranchii	75	24	1.6990	3.2304	10	4000	0.0000	59	descriptive within-class support is much smaller; coefficients are supplementary

The Family-clustered model uses CR2 standard errors with Satterthwaite degrees of freedom. These analyses do not replace the canonical OLS model.
